# Exploring Helical Fraying Linked to Dynamics and Catalysis in Adenylate Kinase

**DOI:** 10.1021/acs.biochem.5c00306

**Published:** 2025-10-03

**Authors:** Jonna Mattsson, Chanrith Phoeurk, Léon Schierholz, Ameeq Ul Mushtaq, Jhon Alexander Rodriguez Buitrago, Per Rogne, A. Elisabeth Sauer-Eriksson, Magnus Wolf-Watz

**Affiliations:** † Department of Chemistry, 8075 Umeå University, 901 87 Umeå, Sweden; ‡ Department of Bio-Engineering, Royal University of Phnom Penh, 120404 Phnom Penh, Cambodia; § Department of Molecular Biology, Umeå University, 901 87 Umeå, Sweden; ∥ Department of Pharmacology, Northwestern University, Feinberg School of Medicine, Chicago Illinois 60611, United States; ⊥ Department of Chemistry, 50106 Universidade Nova de Lisboa, Science and Technology Faculty, Caparica 2829-516, Portugal

## Abstract

Conformational dynamics is a fundamental aspect of enzymatic catalysis that, for example, can be linked to ligand binding and release, assembly of the active site, and the catalytic mechanism. The essential and metabolic enzyme adenylate kinase (AK) undergoes large-scale conformational changes in response to binding of its substrates ATP and AMP. As such, it has been intensely studied in search of linkages between dynamics and catalysis. For a complex conformational change to occur in a protein, whether it is of an induced fit or conformational selection nature, changes at several hinges are often required. Here, based on a comparative structure–function analysis of AK enzymes from *E. coli* and the archaea *Odinarchaeota* and from human AK1, we found that conformational changes in the enzymes are to a varying degree linked to bending, fraying, or unfolding/folding events of the termini of α-helices observed in various structural hot spots of the enzymes. The findings contribute with a mechanistic angle to how enzymatic dynamics and catalysis relate to the plasticity of the termini of α-helices.

## Graphical abstract

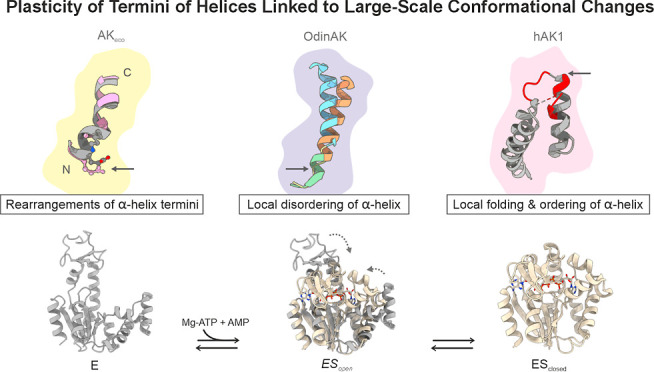

## Introduction

Functional outputs of proteins require time-dependent changes to their structure or assembly. One class of time-dependent changes is the assembly of individual subunits into large macromolecular machines in, for instance, transcription (RNA polymerase^1^), translation (ribosomes^2^), protein folding (GroEL/ES^3^), and proteostasis (proteasome^4^). Assembly of complexes is a process that is dependent on diffusion of proteins to prime interactions through formation of encounter complexes with productive geometries.^5,6^ In contrast to these massive assembly processes, the action of single chain enzymes is often dependent on conformational dynamics,^7^ which is a time-dependent process with a significantly smaller spatial amplitude compared to the assembly of the molecular machines described above. Conformational dynamics is here defined as time-dependent changes to the Cartesian coordinates *x*, *y*, and *z* in the protein structure. For enzymes, the spatial amplitude for dynamics varies and depends on the purpose of the dynamic event. Examples of smaller changes are reorganization in loops that are linked to the catalytic event,^8^ whereas larger changes are, for instance, domain reorientations that occur to enable substrate binding and assembly of active sites.^9^ Conformational dynamics can represent the rate-limiting step in enzymatic reaction cycles^10–12^ and can also be directly linked to catalysis, as has been suggested for phosphatases^8^ and adenylate kinases.^13^ From a macroscopic standpoint, domain and subdomain motions are generally explained with the frameworks of induced fit^14^ and conformational selection models^15–17^ or hybrids thereof.^18,19^ The underlying microscopic events enabling macroscopic transitions in proteins are of diverse nature and include rigid body translations,^20,21^ loop fluctuations,^8^ coupled folding and binding,^22^ order to disorder to order transitions,^23^ and local unfolding or “cracking”.^24–27^ Furthermore, two structures may essentially be the same, differing only by local, small-scale fluctuations such as helical or sheet fraying.^28^

The essential metabolic enzyme AK^29^ is present in all organisms and has emerged as a suitable model enzyme for understanding linkages between conformational dynamics and enzymatic catalysis.^13,30–32^ Key factors making this enzyme suitable for these types of studies include its relatively small size (∼20 kDa) and favorable biophysical properties such as high solubility,^33^ ease of crystallization and structural analysis,^34,35^ and its excellent NMR spectra.^36^ AK undergoes large-scale conformational changes that shuttle the enzyme from the open state to the closed and catalytically active state in response to binding of its substrates ATP and AMP.^35,37^ Previous studies have shown that the large-scale ATP-dependent conformational change linked to the transition from the open to closed state is initiated by a cation–π interaction between an arginine side chain and the adenosine base of ATP,^38^ and that the selectivity toward NTP substrates is controlled with a selectivity loop that can be of varying length in different AKs.^39,40^ Moreover, the catalytic mechanism of AK involves a rate-limiting opening of the substrate binding domains,^12,13^ although alternative mechanisms have been suggested.^41^ The large-scale conformational change of *Escherichia coli* AK (AK_eco_) occurs on hinges that are distributed across the enzyme.^42,43^ In this study, we explore hinges positioned in helices by deploying a comparative structural and functional study of AK from three model organisms. The results suggest that the plasticity of the termini of α-helices is likely a factor in enzymatic catalysis in the studied examples.

## Materials and Methods

### Enzyme Production

Human AK1 cloned in a pET24d expression vector was overexpressed in *Escherichia coli* BL21 (DE3) by adding 1 mM isopropyl β-d-1-thiogalactopyranoside at 18 °C in M9 minimal medium enriched with ^15^NH_4_Cl as the sole nitrogen source (99% ^15^N; Cambridge Isotope Laboratories, Inc., Tewksbury, MA, USA) and in the presence of glucose or ^13^C-labeled glucose as the sole carbon source (99% U-^13^C6; Cambridge Isotope laboratories, Inc.). Harvested cells were suspended in buffer (50 mM Tris-HCl and 1% TritonX-100 at pH 7.5) and lysed by sonication on ice, followed by centrifugation of the lysate at 16,000 rpm for 45 min with a Beckman JA-25.50 rotor. A Blue Sepharose affinity column was used to capture nucleotide binding proteins (including hAK1) in the supernatant obtained after centrifugation, and a linear NaCl gradient was used for elution. Elution fractions containing hAK1 were subjected to size-exclusion chromatography (HiPrep 26/60 Sephacryl S-100 HR; GE Life Sciences) in a buffer consisting of 30 mM MOPS and 50 mM NaCl, pH 7.0. Protein concentrations of hAK1 were determined using an extinction coefficient of 10,430 M^–1^ cm^–1^ at λ = 280 nm. hAK1 was concentrated below 1.5 mM, due to precipitation of protein at higher concentrations, and stored at 4 °C. Protein purification steps were performed using ÄKTA prime and ÄKTA purifier systems (GE Healthcare). The OdinAK and AK_eco_ variants were expressed and purified as previously described in references 40 and 44, respectively.

### NMR Spectroscopy

All NMR spectra were recorded on Bruker AVANCE III HD 850 and 600 MHz spectrometers using a triple-resonance (TXI 5 mm) cryoprobe equipped with pulsed field gradients along the *x*, *y*, and *z* axes. For the AK_eco_ variants, 2D NMR ^1^H–^15^N-HSQC spectra were acquired on ^15^N-labeled protein at a concentration of 0.9 mM and 0.6 mM for the Lys47Ala and Glu114Ala variant, respectively. The sample buffer consisted of 30 mM MOPS, 50 mM NaCl at pH 7.0, with 7% (v/v) D_2_O added. The 2D ^1^H–^15^N HSQC experiments were performed using 4 number of scans, time-domain sizes of 256 (^15^N) × 2048 (^1^H) complex points, and sweep widths of 10869.565 Hz (^1^H) and 3015.391 Hz (^15^N). In the case of backbone assignments, a series of triple-resonance experiments using HNCA, HN­(CO)­CA, HNCACB, CBCA­(CO)­NH, HNCO, and HN­(CA)­CO were performed at 298 K using uniformly ^13^C/^15^N-labeled 0.8 mM human AK1 in 30 mM MES, 50 mM NaCl buffer at pH 5.5, and D_2_O 10% (v/v) was added to all NMR samples for the field-frequency lock. All 2D ^1^H–^15^N HSQC experiments were performed using 8 or 16 number of scans, time-domain sizes of 256 (^15^N) × 2048 (^1^H) complex points, and sweep widths of 10204.08 and 2411.96 Hz along the ^1^H and ^15^N dimensions, respectively. NMR spectra were processed using NMRPipe and NMRDraw software^45^ and visualized and analyzed using Topspin 3.6 (Bruker), CCPN2.1.5,^46^ and Sparky (v3.113; https://www.cgl.ucsf.edu/home/sparky; UCSF, San Francisco, CA, USA). Sequential backbone-NMR assignments for ^15^N, ^13^Cα, ^13^Cβ, ^13^C′, ^1^Hα, ^1^Hβ, and ^1^H_N_ atoms were performed by correlating resonances according to their inter-residue ^13^C and ^1^H correlations. Assigned chemical shifts were directly referenced against 4,4-dimethyl-4-silapentane-1-sulfonic acid for the ^1^H atoms, whereas ^13^C and ^15^N atoms were referenced indirectly as suggested (http://www.bmrb.wisc.edu). Secondary structure predictions were performed with TALOS + using ^1^H_N_, ^15^N, ^13^Cα, ^13^Cβ, and ^13^C chemical-shift data.^47^

In order to analyze the parameters associated with ^15^N-relaxation, interleaved 2D NMR experiments based on ^1^H–^15^N correlation spectra were collected for T_1_, T_2_, and ^1^H–^15^N-heteronuclear nuclear Overhauser effects (NOEs). Delays of 50, 100 (duplicate), 200, 400, 500, 600, 800, 1000 (duplicate), 1200, and 1500 ms were used for *T*_1_ measurements, and 16.96, 33.92, 50.88, 84.80, 101.76, 118.72, 135.68, 152.64, 169.60 (duplicate), 186.56, 203.52, 254.40, and 288.32 ms were used for *T*_2_ measurements, as described previously.^48^ Relaxation delays of 3 and 2 s were used for *T*_1_ and *T*_2_ experiments, respectively. Steady-state ^15^N–^1^H-heteronuclear NOE spectra were measured with either 5 s delays between each free-induction decay or 2 s delays, followed by a 3 s series of 120° nonselective ^1^H pulses as previously described.^49^ *T*_1_, *T*_2_, and ^15^N–^1^H NOE experiments were performed with time-domain sizes of 256 × 2048 complex points and sweep widths of 10204.08 and 2411.96 Hz along the ^1^H and ^15^N dimensions, respectively, with 8 or 16 scans for *T*_1_ or *T*_2_ and 40 scans for the ^15^N–^1^H NOE experiment.

### Analysis of ^15^N-Relaxation Data

SPARKY (v3.113; https://www.cgl.ucsf.edu/home/sparky; UCSF, San Francisco, CA, USA) was used to analyze the ^15^N-relaxation data of human AK1 states. Peak heights of the ^1^H–^15^N cross-peaks in the *T* ^1^ and *T* ^2^ spectra were measured using a peak-picking routine in SPARKY and then fitted to a single exponential-decay function using the Curvefit module in SPARKY

1 $$I\left(t\right)=I_{0}\times e^{\left(-t/Td\right)}$$

where *I*(*t*) represents the intensity of the signal at time *t*, *I*_0_ represents the intensity at time *t* = 0, and *T*_d_ represents the decay constant for *T*_1_ or *T*_2_. Errors in *T*_1_ and *T*_2_ were estimated from the fittings by using 500 Monte Carlo simulations. ^15^N–^1^H-heteronuclear NOE values were calculated from the ratio of peak intensities, *I*_sat_/*I*_unsat_, where *I*_sat_ and *I*_unsat_ represent the intensities of peaks in saturated and unsaturated spectra, respectively. The NOE error (σ_NOE_) was calculated as

2 $$\sigma_{NOE}=\frac{I_{sat}}{I_{unsat}}{\left[{\left(\frac{\sigma_{sat}}{I_{sat}}\right)}^{2}+{\left(\frac{\sigma_{unsat}}{I_{unsat}}\right)}^{2}\right]}^{1/2}$$

where σ_sat_ and σ_unsat_ represent the root-mean-square variation in the noise in empty spectral regions of the spectra in the presence and absence of proton saturation, respectively.

### Model-Free Analysis

Internal motion parameters were determined according to model-free formalism^50,51^ using ROTDIF3 software.^52,53^ Rotational diffusion tensors of apo, ADP, and Ap5A states of human AK1 molecules (PDB IDs: 2C95 (with ligand removed), 2C95, and 1Z83) were estimated from the ^15^N spin-relaxation data acquired at a 850 MHz field. The axially symmetric model was shown to best fit the experimental data of human AK1 states. Relaxation data were optimized to the diffusion model using 500 Monte Carlo simulations and assuming an internuclear distance (*r*_NH_) of 1.02 Å and chemical-shift anisotropy of −160 ppm for the ^15^N nucleus. The overall motion of the protein in the isotropic diffusion model is characterized by the overall correlation time, τ_m_, and its spectral-density function is represented as

3 J(ω)=S2τm(1+ω2τm2)+(1−S2)τ(1+ω2τ2)

with

$$\frac{1}{\tau }=\frac{1}{\tau_{m}}+\frac{1}{\tau_{e}}$$

where τ_e_ represents the correlation time for internal motions and *S* ^2^ = *S*_*f*_^2^ *S*_*S*_^2^ represents the generalized order parameter. *S*_*f*_^2^ is the generalized order parameter for fast internal motion on the sub-ns time scale and *S*_*S*_^2^ is the order parameter for slow internal motion on the ns time scale.^54^

After deriving a global τ_m_, *S* ^2^ and τ_e_ were fitted against the experimental data until an acceptable solution was obtained by minimization of the target function, χ^2^.^55^

4 $$\chi^{2}=\sum \left[\left(T_{1}^{\exp}-T_{1}^{cal}\right)/\sigma_{1}^{2}+\left(T_{2}^{\exp}-T_{2}^{cal}\right)/\sigma_{2}^{2}+\left({NOE}_{2}^{\exp}-{NOE}_{2}^{cal}\right)/\sigma_{N}^{2}\right]$$

In this equation, the summation extends over all of the amino acid residues, with the superscripts indicating either experimental or calculated values and sigma representing the standard error of the parameter.

### Enzymatic Activity Measurements

Enzymatic activities of the OdinAK and AK_eco_ enzymes were determined either by an ATPase assay^56^ or a ^31^P real-time NMR spectroscopy assay.^57^ The enzymatic activity of the AK_eco_ variants was quantified with the coupled ATPase assay as previously described.^39^ In short, the ADP production was coupled to the oxidation reaction of NADH by the coupling enzymes pyruvate kinase and lactic dehydrogenase, and the change in absorbance at 340 nm was continuously followed. In order to determine *K*_M_ and the reaction velocity at saturating substrate conditions (*k*_cat_), the Michaelis–Menten equation (eq 5) was fitted to the normalized reaction velocities (*V*/[*E*]_tot_) against increasing substrate concentrations ([*S*]). Experimental errors were estimated by technical triplicates.

5 $$\frac{V}{{\left[E\right]}_{tot}}=\frac{k_{cat}\left[S\right]}{\left(K_{M}+\left[S\right]\right)}$$

To determine the enzymatic activity of the OdinAK Ser74Gly variant, a real-time ^31^P NMR spectroscopy activity assay^57^ was instead used. The reaction mixture contained 1 mM ATP, 300 μM AMP, 5 mM MgCl_2_, BSA (0.2 mg/mL) and 7% D_2_O (v/v) in 30 mM MOPS, 50 mM NaCl at pH 7.0, and the reaction was initiated by adding OdinAK Ser74Gly to a final concentration of 1 μM or 50 nM depending on the set experimental temperature. A series of one-dimensional (1D) ^31^P NMR spectra were acquired at 298 K at constant time intervals where the buildup of ADP was followed. The NMR data were acquired on a 600 MHz Bruker AVANCE III HD spectrometer equipped with a broadband observe (BBO) cryoprobe. The resulting peaks were integrated with TopSpin 3.6.3 (Bruker) to determine the concentrations of the mono-, di-, and triphosphates. The obtained concentrations were plotted against the reaction time, and the data were fitted using OriginPro 2020 (OriginLab) as described in ref 57 in order to quantify the catalytic efficiency (*k*_cat_). Three technical replicates were conducted for error estimates.

### Circular Dichroism Spectroscopy

Thermal unfolding experiments were performed on a Jasco J-810 spectropolarimeter in a 1 mm cuvette. The protein concentration was 15 μM in buffer consisting of 30 mM MOPS, 50 mM NaCl at pH 7.0 for both AK_eco_ variants. For the inhibitor bound thermal unfolding experiments, Ap5A was added in a 10-fold stoichiometric excess. Thermal unfolding was monitored at a wavelength of 220 nm simultaneously as the temperature was raised from 20 to 80 °C at a rate of 1 deg min^–1^. The melting point (*T*_m_) was quantified by nonlinear fits of the obtained data to a two-state transition in the OriginPro 2020 (OriginLab).^58^

### Protein Crystallization

All crystals were obtained at 18 °C by using the sitting-drop vapor-diffusion method. The AK_eco_ Glu114Ala variant in complex with Ap5A (molar ratio 1:2) was crystallized at a protein concentration of 15 mg/mL (in buffer 30 mM MOPS, 50 mM NaCl at pH 7.0). The crystallization drops consisted of 1 μL of protein preincubated with Ap5A, mixed with 1 μL of the precipitant solution (0.2 M sodium acetate, 0.1 M sodium citrate tribasic dihydrate pH 4.6, and 32% (w/v) polyethylene glycol (PEG) 4000). For the AK_eco_ Lys47Ala variant, crystallization drops consisted of 1 μL protein (20 mg/mL) preincubated with Ap5A (molar ratio 1:2), mixed with 1 μL precipitant solution (0.2 M ammonium sulfate, 0.1 M sodium acetate pH 5.4, and 30% (w/v) PEG 4000). The crystals of both AK_eco_ variants were cryoprotected in 25% (v/v) glycerol before being flash frozen in liquid nitrogen. Crystals of the OdinAK Ser74Gly variant (protein concentration 10 mg/mL) grew in 0.2 M magnesium acetate tetrahydrate, 0.1 M sodium cacodylate trihydrate pH 6.5, and 20% (w/v) PEG 8000 and were cryoprotected in 25% (v/v) glycerol before flash freezing in liquid nitrogen. X-ray diffraction data were collected at 100 K on beamlines ID23-2 and ID30A-3 at the European Synchrotron Radiation Facility laboratory (Grenoble, France).

### Structure Determination and Refinement

The obtained diffraction images of the AK_eco_ Glu114Ala and Lys47Ala variants were processed using the CCP4 suite.^59^ The crystallographic structures of the AK_eco_ variants were solved by molecular replacement using either PHASER or MrBUMP,^60,61^ in both instances with the structure of AK_eco_ in complex with Ap5A (PDB ID: 1AKE) as a search model. The structures were refined using REFMAC^62^ combined with manual model rebuilding using COOT.^63^ The structure of the OdinAK Ser74Gly variant was determined by molecular replacement with the apo OdinAK structure (PDB ID: 7OWH) as a search model. Structural modeling and refinement of OdinAK were done using COOT and PHENIX REFINE.^64^ For data collection and refinement statistics of the AK_eco_ and OdinAK structures, see Table S1.

## Results and Discussion

AKs contain distinct structural elements and loops that are integral to their function. In addition to the well-characterized ATP- and AMP-binding domains (ATPlid and AMPbd, respectively), the enzyme has three functional loop segments including the p-loop,^65^ the selectivity loop,^39^ and the catalytic loop.^13^ We have performed our studies on AKs from bacteria (*Escherichia coli*; AK_eco_), archaea (*Odinarchaeota*; OdinAK), and eukarya (*Homo sapiens*; hAK1). The specific enzymes are representatives from the three domains of life and are well-characterized from both a structural and functional standpoint.^25,40,66–68^ Figure 1 shows the structures of the selected AK enzymes in their closed conformation with their ATPlid and AMPbd, along with the functional loops, highlighted.

**Figure 1:**
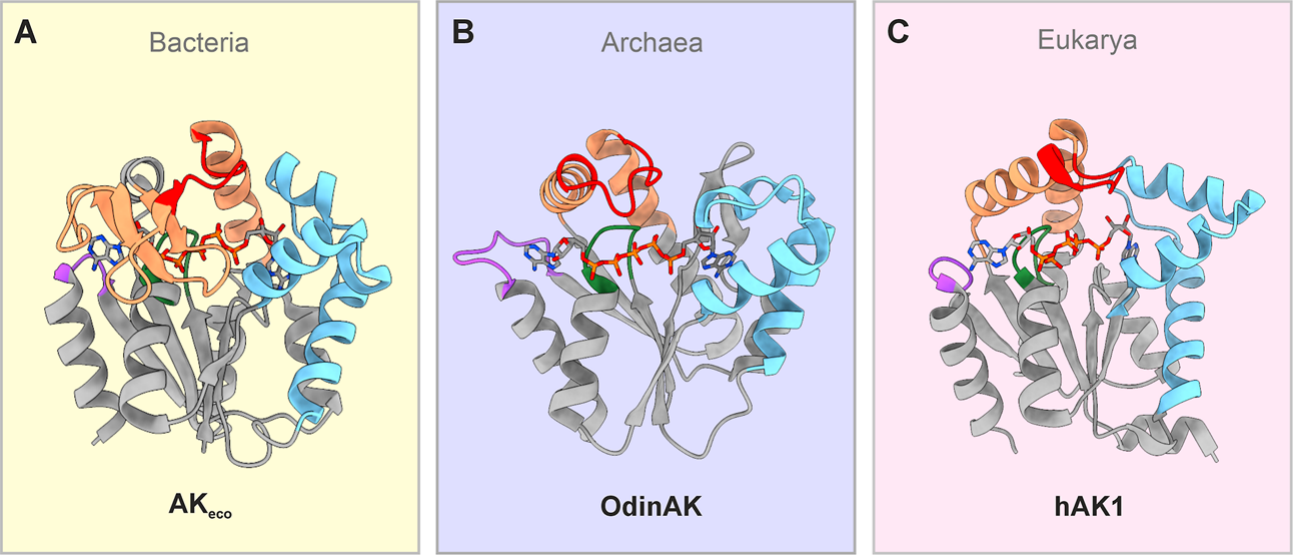
Ribbon representation of model AK enzymes in their closed conformations. (A) AK_eco_ in complex with the inhibitor P1,P5-di­(adenosine-5)­pentaphosphate (Ap5A) (PDB ID: 1AKE).^35^ (B) monomer A of trimeric OdinAK in complex with Ap5A (PDB ID: 7OWE).^40^ Figure S1 shows the biological trimer of OdinAK. (C) hAK1 in complex with Ap5A (PDB ID: 1Z83 (unpublished)). Color-coding is as follows: ATPlid in orange (residues Asp113–Thr175 in AK_eco_), AMPbd in blue (residues Thr31–Glu75), p-loop in green (residues Gly7–Lys13), catalytic loop in red (residues Thr154–Glu161), and the selectivity loop in purple (residues Asp197–Pro201).

### Helical Elements Involved in Dynamics Underlying Subdomain Closure in Bacterial Adenylate Kinase

AK_eco_ has emerged as one of the principal model systems to disentangle the linkages between enzyme-structure, stability, dynamics, and catalysis.^7,32,34,69^ This is reflected in the large number of mechanistic studies of the enzyme that have been undertaken with a broad range of experimental and computational approaches. These include single molecule FRET microscopy,^41,70^ single molecule nanopore spectroscopy,^71^ X-ray crystallography,^37,72^ NMR spectroscopy,^36,66,69,73^ and MD-^74^ and QM/MM simulations.^13^ It has been proposed that local unfolding/refolding events are key elements for the large conformational change involved in the open-to-closed structural transition in AK_eco_.^25,26,75^ These microscopic behaviors have been formulated in a concept described as “cracking“.^24^ Local unfolding in AK_eco_ has also been proposed to link dynamics with evolved thermal adaptation.^76^ Inspired by the roles of local folding and unfolding events, we made a careful comparison of local structural differences between AK_eco_ in its open (apo) and closed (complexed with Ap5A) states. Based on the analysis, we identified two α-helices denoted the E114-helix and K47-helix that display changes on their open-to-closed conformational trajectory (Figure 2A,B). These α-helices transition from regular straight helices into helices including a significant bend that appears to be linked to interactions in the termini of the helices themselves. Changes in the E114-helix (residues Asp113–Gly122) located in the ATPlid are linked to changes in the interaction pattern for residue Glu114 (Figure 2C). In the open state, this residue forms a salt bridge to the side chain of Arg165 positioned on a separate helix. Following closure of the ATPlid and AMPbd, the salt bridge is broken, and the side chain of Glu114 changes rotamer, enabling the formation of a hydrogen bond to its backbone amide proton. This results in a stabilizing N-cap-like interaction, which causes the N-terminal end of the E114-helix to bend (Figure 2C). Two helix-integrated water molecules form water-mediated hydrogen bonds within the helix that stabilize the bent helical structure (Figure 2C). The K47-helix (residues Glu44–Gly56) is located in the AMPbd (Figure 2D). Compared to the E114-helix, the K47-helix undergoes more of an opening of the N-terminal end of the helix on the open-to-closed trajectory. In the open state, the K47-helix exists as a straight α-helix with no interaction partners to the side chain of Lys47. In the closed state, however, the side chain of Lys47 shifts rotamer and forms an electrostatic interaction with the side chain of Glu44 located at the N-terminus of the helix. In addition, the side chain of Gln48 changes rotamer and distorts the N-terminal of the helix by making hydrogen bonds to main chain atoms. Combined, these structural changes cause the first turn of the K47-helix to open up (Figure 2D). Overall, the common feature for both the E114- and K47-helices seems to be the conformational plasticity at their N-termini, which in part appears to depend on a local interaction pattern and therefore on local thermodynamic stability at their respective terminal positions. In order to test the importance of these local interactions in the N-terminus, we perturbed the AK_eco_ enzyme by replacing Glu114 and Lys47 individually with alanine (Glu114Ala and Lys47Ala variants, respectively) and monitored changes to both structure and catalysis. In both cases, substitutions were made to alanine in order to maintain the intrinsic stability of the helices.^77,78^

**Figure 2:**
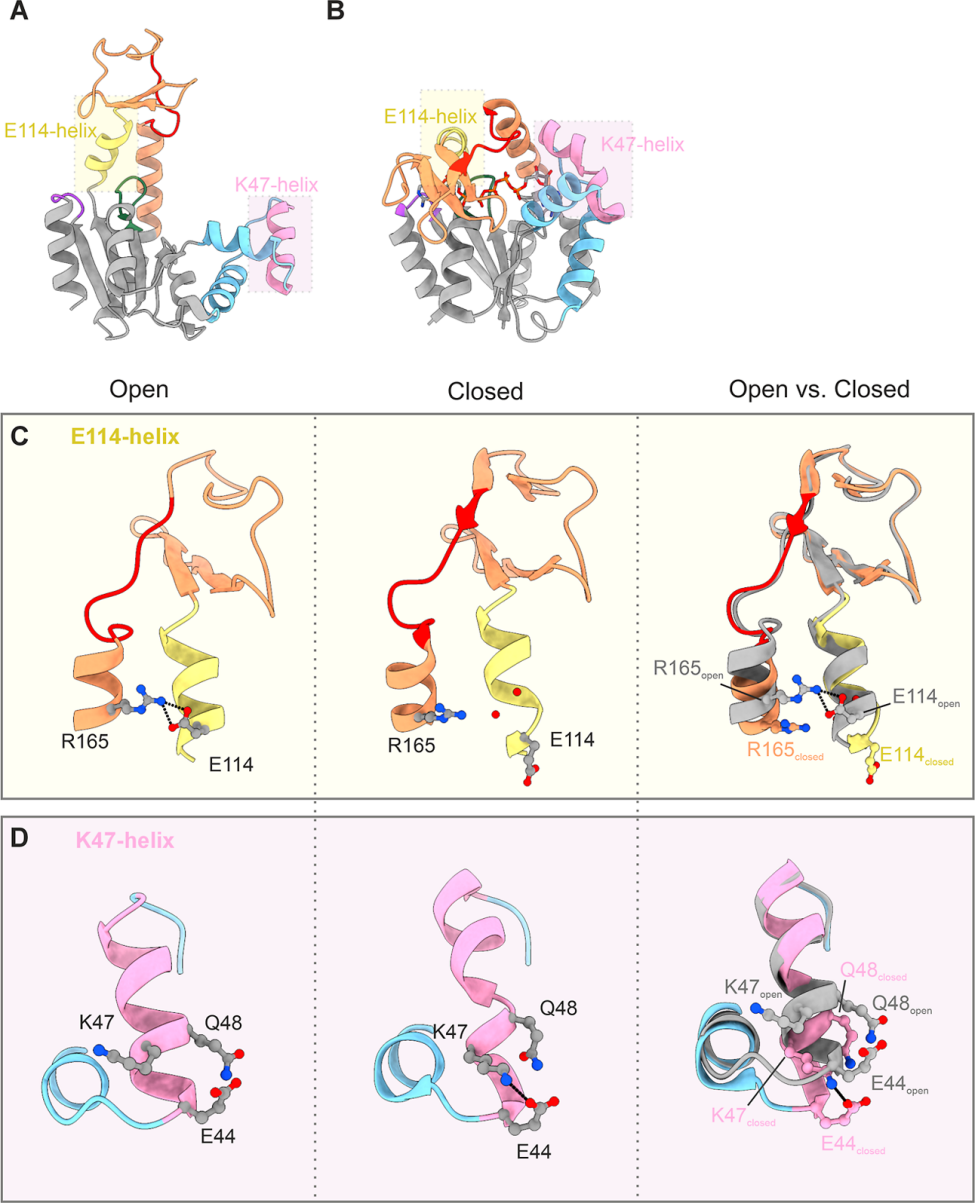
Superposition of E114- and K47-helices in open and closed states highlights the change in local helical geometry of the open-to-closed transition. (A) Chain A of apo AK_eco_ (PDB ID: 4AKE)^37^ color-coded as in Figure 1 but with the E114-helix in yellow and the K47-helix in pink. (B) Chain A of closed Ap5A-bound AK_eco_ (PDB ID: 1AKE). (C) The E114-helix with residues Arg165 and Glu114 shown as sticks in the open and closed states, left and middle panels, respectively. Superposition of the E114-helices in apo (gray) and Ap5A-bound states (yellow) revealed fraying and bending of the helix in the closed state (right panel). The superposition is based on residues Leu115–Asp158 (main chain atoms). Two water molecules integrated in the E114-helix in the closed structures are shown as red spheres. (D) The K47-helix with residues Glu44, Lys47, and Gln48 shown as sticks in the open and closed states, left and middle panels, respectively. Superposition of the K47-helices in apo (gray) and Ap5A-bound states (pink), right panel. The superposition is based on residues Gln48–Asp61.

### Destabilization of the E114-Helix in the Glu114Ala Variant Affects Catalysis of AK_eco_

The structure of the AK_eco_ Glu114Ala variant in complex with the inhibitor Ap5A was determined to 1.6 Å resolution with X-ray crystallography (Table S1). In the Glu114Ala variant, the ATPlid and AMPbd subdomains are, as expected, positioned in a closed conformation, identical to the Ap5A-bound native AK_eco_ structure (Figure 3A). The observed salt bridge between Glu114 and Arg165 present in the open state of the AK_eco_ structure is disrupted in the Glu114Ala variant, and there are no significant structural effects on the overall structure due to the Glu to Ala substitution in the closed state. However, a close-up view of the N-terminus of the E114-helix in the closed state revealed two bound water molecules that seem to compensate for the missing carboxyl group of the Ala114 side chain (Figure 3B,C).

**Figure 3:**
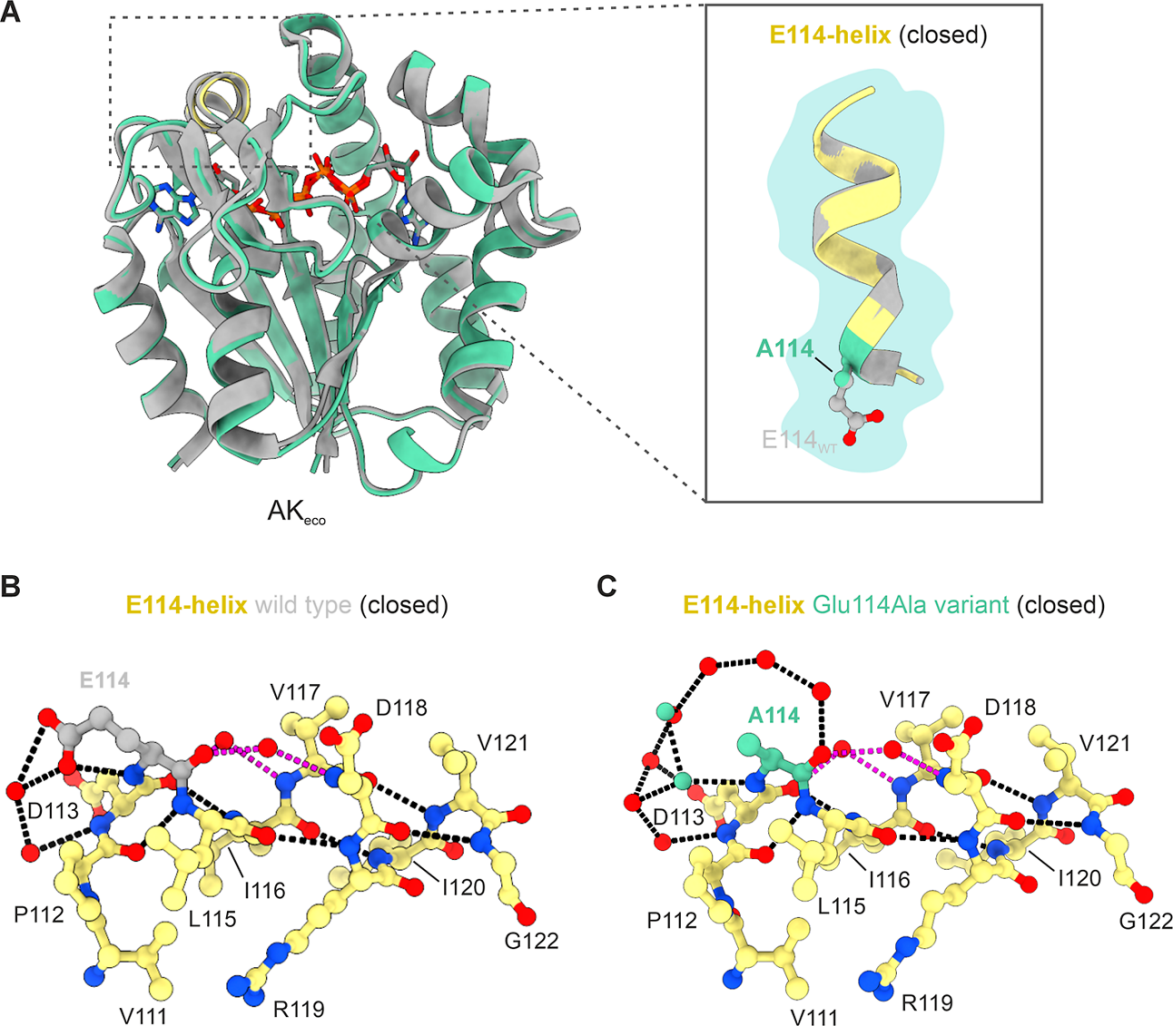
Structure of the AK_eco_ Glu114Ala variant in complex with Ap5A. (A) Overlay of chain A of native AK_eco_ (gray, PDB ID: 1AKE) and the Glu114Ala variant (turquoise, PDB ID: 9R71 (this study)) in complex with Ap5A. The E114-helix is highlighted in yellow in the Glu114Ala structure. Superposition of the main chain atoms of all residues (Met1–Gly214) gave a root-mean-square deviation (rmsd) of 0.4 Å. The right panel shows a zoom-in on the E114-helix (residues Asp113–Gly122) for native AK_eco_ (gray) and the Glu114Ala variant (yellow). The side chains at position 114 are shown in both the native (gray) and Glu114Ala variant (turquoise) structure as sticks. (B) Stick representation of the E114-helix (residues Asp113–Gly122) in wild-type AK_eco_ with Glu114 highlighted in gray. Hydrogen bonds within the helix and to a selection of water molecules are shown as black dotted lines. Hydrogen bonds to two integrated and helix-bending water molecules are shown in magenta. (C) Stick representation of the E114-helix in the Glu114Ala variant in the same orientation as in (B), with Ala114 highlighted in turquoise. The mutant variant displays additional water molecules in the first hydration shell around the helix. Two water molecules in the Glu114Ala variant, shown here in turquoise, occupy the position of the oxygen atoms in the native Glu114 carboxyl group.

The Arg165–Glu114 salt bridge most likely stabilizes the ATPlid in the open state, and removal of this interaction could therefore potentially destabilize the ATPlid and affect the structural equilibrium. As such, the Glu114Ala variant is expected to shift its conformational equilibrium toward a closed state. This hypothesis was strengthened by the determined catalytic activity, where *K*_M_ of the AK_eco_ Glu114Ala variant is reduced approximately 3-fold compared to wild-type AK_eco_ (Table 1). This indicates a higher affinity of the Glu114Ala variant for its substrate. Relative to native AK_eco_, the *k*_cat_ for the Glu114Ala variant is only slightly decreased and in combination with the reduced *K*_M_, the specificity constant is increased more than 2-fold.

**Table 1:** Catalytic Parameters of AK_eco_ Variants.

	*k*_cat_ (s^–1^)	*K*_M_ (μM)	*k*_cat_/*K*_M_ (μM^–1^ s^–1^)
wild type	360 ± 11^a^	110 ± 9	3.3 ± 0.3
Lys47Ala	464 ± 3	116 ± 8	4.0 ± 0.3
Glu114Ala	254 ± 4	36 ± 7	7.1 ± 1.3

a Errors are estimated from fits of *k*_cat_ and *K*_M_ to eq 5 (fits are found in Figure S2) from three technical replicates.

The thermal stability of the Glu114Ala variant was evaluated by circular dichroism (CD) spectroscopy thermal unfolding experiments. We found that the Glu to Ala substitution decreased the melting temperatures (*T*_m_) with 3.4 °C in the open state and 1.8 °C in the closed Ap5A-bound state, compared to wild-type AK_eco_ (Table S2 and Figure S3). Thus, the thermal stability data show that the Glu to Ala replacement affects the open state. This is consistent with the observed reduction in *k*_cat_ being linked to the stability of the N-terminus of the helix (i.e., fraying). It is possible, however, that the small observed destabilization in the closed state is due to the increased binding affinity (reduced *K*_M_) as determined for the Glu114Ala variant and relative to the wild type. In an attempt to observe potential perturbations of the equilibrium between open and closed states in response to the Glu114Ala substitution, we analyzed the degree of closure of the enzyme variant by quantitative analysis of chemical shifts.

We used so-called projection analysis applied to chemical shifts^79^ to probe whether the Glu114Ala replacement perturbs the open-to-closed equilibrium of the enzyme. The used approach and analysis are described in detail in the Supporting Information. In short, the approach and analysis are each based on the definition of a chemical shift vector sensitive to the open-to-closed conformational transition. This vector is constructed from the open (substrate-free) to the closed (Ap5A-bound) state for wild-type AK_eco_. Onto this reference vector, vectors sensitive to the open-to-closed transition for the substrate-free and Ap5A-bound states of the Glu114Ala variant are projected. The projections provide two parameters: a directionality of the chemical shift change for the variant relative to that of the wild type (cosθ) and the degree of closure relative to that of the wild type (*X*).^79^ For the Glu114Ala variant in complex with Ap5A, the majority of residues has a directionality of the chemical shifts that superimpose with those of the wild type (i.e., cos­(θ) > 0.9), and of these residues, the vast majority has an open-closed equilibrium (*X* = 1) that is on par with that of the wild type (Figures S4 and S6). Therefore, the global open-to-closed equilibrium in the Ap5A-bound Glu114Ala variant is of the same magnitude as the wild type within the experimental uncertainty. Some residues do, however, exist with values of *X* significantly larger or smaller than one, and we interpret these as local fluctuations that possibly could affect enzymatic catalysis.

By performing the same analysis on the open state of the Glu114Ala variant (Figure S7), we found only a small subset of residues with a directionality that superimposes with the open-to-closed transition for the wild type (i.e., cos­(θ) > 0.9). Since there exists a significant spread in the parameter cosθ, it is possible that several dynamic modes exist for the substrate-free Glu114Ala variant. For the residues with a cos­(θ) > 0.9, the average value of closure relative to the wild type (*X*) is low (0.13 ± 0.15, the large standard deviation is likely due to the contribution of other dynamic effects of similar magnitude) suggesting a minute shift toward the closed state for this dynamic mode. Thus, it appears that the dynamic landscape of the open state of the Glu114Ala variant has changed relative to the wild type and that only one of several dynamic modes corresponds to sampling of the open-closed equilibrium. For both the open and closed states, the analysis suggests that the main difference relative to the wild type is that the open state of Glu114Ala seems more engaged in a number of dynamic modes, one of which likely corresponds to the open-to-closed equilibrium. However, it is not possible to correlate these data directly with the observed reduction of catalytic activity in the Glu114Ala variant. Taken together, the data agree with the Glu114-Arg165 salt bridge being important for the inherent local structural stabilization of the open conformation of the ATPlid in AK_eco_.

### The Plasticity of the K47-Helix Affects the Catalytic Turnover Rates in AK_eco_

The crystallographic structure of the AK_eco_ Lys47Ala variant in complex with the inhibitor Ap5A was determined to 1.8 Å resolution (Table S1). As expected, this AK_eco_ variant adopts a structure nearly identical to the native structure and has the ATPlid and AMPbd subdomains in the closed conformation (Figure 4A). Structural comparison of the K47-helix in the Lys47Ala variant and in the native Ap5A-bound structure shows that there are no major changes to the helical structure (Figure 4A). In addition, the overall and global conformation of the AMPbd, key for selective binding to AMP, remains intact in the Lys47Ala variant. The observed local helical distortion caused by the side chain of Gln48 binding to the main chain nitrogen atom of Gly45 is further maintained in the Lys47Ala structure (Figure 4B,C).

**Figure 4:**
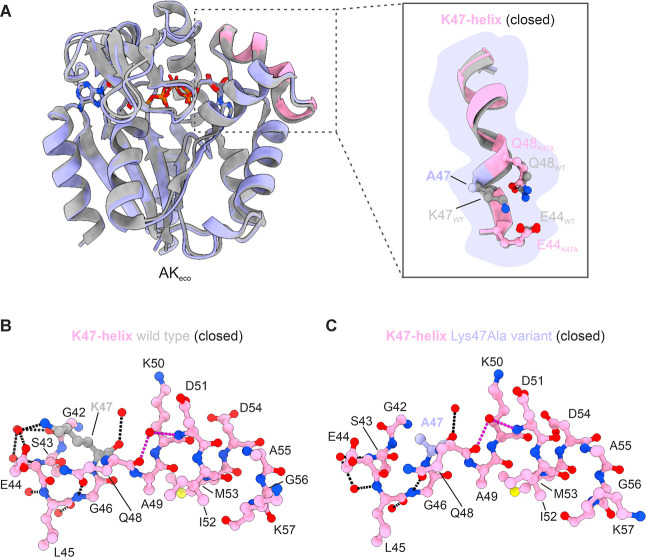
Structure of the AK_eco_ Lys47Ala variant in complex with Ap5A. (A) Overlay of chain A of native AK_eco_ (gray, PDB ID: 1AKE) and the Lys47Ala variant (purple, PDB ID: 9R6U (this study)) in complex with Ap5A. The K47-helix is highlighted in pink in the Lys47Ala structure. Superposition of the main chain atoms of all the residues (Met1–Gly214) gave a rmsd of 0.4 Å. The right panel shows a zoom-in on the K47-helix (residues Glu44–Gly56) for native (gray) and the Lys47Ala variant (pink). The side chains at positions Glu44, Lys47, and Gln48 in both the native (gray) and Lys47Ala variant (pink and purple) are shown as sticks. (B) Stick representation of the K47-helix (residues Gly42–Lys57) in native AK_eco_ with Lys47 highlighted in gray. Hydrogen bonds within the helix and to a selection of water molecules are shown as black dotted lines. Hydrogen bonds to one integrated and helix-bending water molecule are shown in magenta. (C) Stick representation of the K47-helix in the Lys47Ala variant in the same orientation as (B) and with Ala47 highlighted in purple.

From a functional perspective, the Lys47Ala variant displays a significant increase in *k*_cat_ relative to the wild type (1.3-fold increase from 360 to 464 s^–1^), while *K*_M_ values for the substrates ATP and AMP remain unchanged (Table 1). This increase in *k*_cat_ is mirrored by an increase in the specificity constant of the Lys47Ala variant by a factor of 1.22.

To investigate a possible link between thermal stability and the change of catalytic parameters, we probed the melting temperatures (*T*_m_) of open and closed states of the Lys47Ala variant with CD spectroscopy. The *T*_m_ of the Lys47Ala variant in apo and Ap5A-bound states decreased with 3.4 and 3.0 °C, respectively, relative to the determined melting points for wild-type AK_eco_ (Table S2 and Figure S3). These differences are significant, and the roughly equal degree of destabilization of the open and closed states indicates that the open-to-closed equilibrium is not affected in the Lys47Ala variant relative to that in the wild type. However, the local destabilization of the K47-helix resulting from the Lys to Ala replacement does correlate with an increased *k*_cat_ value as observed for the Lys47Ala variant. As for the Glu114Ala variant, we attempted to probe changes to the open-closed equilibrium for the Lys47Ala variant by quantifying chemical shifts changes. For the Ap5A-bound state, the majority of residues has an open-closed equilibrium equivalent to the wild type (*X* = 1) (Figures S5 and S8). For the open state, there are changes in dynamics relative to the wild type, and like for the Glu114Ala variant, there appears to be more than one dynamic mode (Figure S9). One of these dynamic modes likely corresponds to an open-to-closed equilibrium shifted toward the closed state relative to that of the wild type (average *X* = 0.11 ± 0.07). For the Lys47Ala variant, there are seemingly changes in the dynamic positions of the K47A-helix, which are potentially linked to the increased *k*_cat_ value compared to the wild type.

### Local Unfolding Event in Subdomain Closure of an Archaeal Adenylate Kinase

The archaeal phylum of the Asgardarcheota, consisting of *Loki*-, *Thor*-, *Heimdall*-, and *Odinarchaeota*, has been suggested to represent the closest known ancestor to eukaryotic cells.^80,81^ We have previously reported structures of open and closed (Ap5A-bound) AK from the archaeal organism *Odinarchaeota* (OdinAK).^40^ Like other archaeal AKs (for instance, *Methanococcus* ^82^), OdinAK oligomerizes into a trimeric conformation that has a large stabilizing effect on the thermostability, resulting in a melting point of 95 °C. This extreme melting point renders the enzyme functional at the very high temperatures found in the habitat of *Odinarchaeota*, the black smokers at the arctic midocean ridge.^83^ Contrarily to bacterial, long AKs, the archaeal AKs have a notably shorter ATPlid and are therefore classified as short AKs.^84,85^ From a functional standpoint, the catalytic activity of the OdinAK trimer is within error compared to an engineered monomeric variant.^40^ To this end, we wanted to examine local structural differences between monomers of OdinAK in the open and closed states. An overlay of OdinAK is shown in Figure 5A where the enzyme displays the hallmark open-to-closed transition seen for AK enzymes. A notable and distinct feature between the native and Ap5A-bound forms is present in the AMPbd, where the last three residues Ile73, Ser74, and Leu75 in the α-helix spanning residues Arg59–Leu75 change to a flexible conformation when bound to Ap5A (Figure 5B). This local unfolding (or disordering) transition is accompanied by a 21° shift of the helix orientation together with a close to 6 Å spatial translation of the helix (Figure 5C). These changes enable the closing of the AMPbd over its substrate. Hence, it seems like a disordering/unfolding event in the C-terminus of the α-helix Arg59–Leu75 (here denoted the S74-helix) is needed for function. One key interaction site to maintain the helical form of residues Ile73–Leu75 is a network of hydrogen bonds between the side chain of Arg2 and Ser74, as well as to the main chain carbonyl oxygens of Ser74, Thr77, Asp78, and Ile106. Many of these interactions are missing in the Ap5A-bound form (Figure 5D). To test whether further destabilization of the helical nature of the segment, by itself, can drive closing of the AMPbd and modulate the activity of OdinAK, we substituted Ser74 for a glycine residue. We chose glycine in order to release the number of hydrogen bonds to Arg2, and since it has a sizable destabilizing effect in α-helices that has been estimated at around 4 kJ mol^–1^ for internal positions relative to alanine.^77,86^ We determined the crystallographic structure of the OdinAK Ser74Gly variant and quantified its catalytic activity with ^31^P NMR spectroscopy.

**Figure 5:**
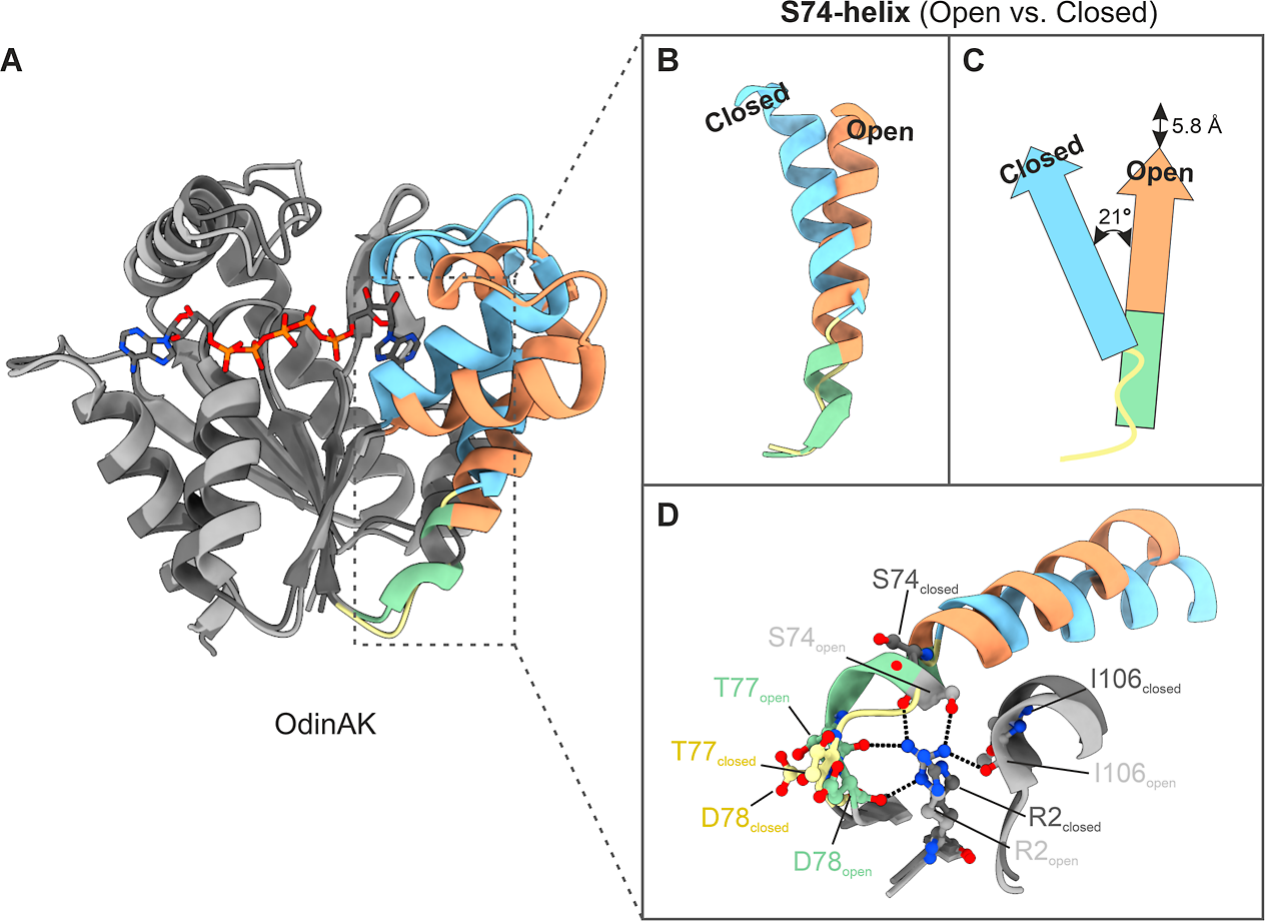
A local unfolding event facilitates the AMPbd closure in OdinAK. (A) Overlay of representative chain A for apo (light gray) and Ap5A-bound (dark gray) states of OdinAK (PDB IDs: 7OWH and 7OWE, respectively^40^). The superposition is based on residues Asp78–Phe196. The AMPbd is colored in orange and in blue for apo and Ap5A-bound states, respectively. The segment spanning residues Ile73–Leu78 including the last helical turn (Ile73–Leu75) of the S74-helix (in the open (apo) state shown in green) undergoes a local unfolding transition upon closure and Ap5A binding (in the closed Ap5A-bound state shown in yellow). (B) Zoom-in on the S74-helix that undergoes the local unfolding event during the open-to-closed transition (color-coded as in panel A). (C) Schematic illustration of the open-to-closed transition that involves a 5.8 Å translation of the S74-helix together with a 21° tilt in the helix orientation. (D) Close-up view of the S74-helix in OdinAK (color-coded as in panel A) with the side chains of the hydrogen bond network residues Ser74, Thr77, Asp78, Ile106, and Arg2 shown as sticks. Hydrogen bonds to the side chain of Arg2 in the apo structure (light gray) are shown as black dotted lines.

### Exploring Local Unfolding in OdinAK Linked to Subdomain Closing

The crystallographic structure of the OdinAK Ser74Gly variant in its substrate-free (apo) form was determined to be 3.6 Å (Table S1). Despite the relatively low resolution, it is clear that the overall fold of the Ser74Gly variant adopts the expected conformation with the AMPbd in a typical open conformation (Figure 6A). In comparison to the native OdinAK structure, there are only minor structural differences near the Ser to Gly substitution. A glycine at position 74 seems to preserve the overall conformation of the S74-helix and does not initiate the unfolding and translational event that is observed in the open-to-closed trajectory in native OdinAK (Figures 5A,B and 6B). The catalytic (*k*_cat_) activity of the Ser74Gly variant was found to be 0.1 ± 0.003 s^–1^ at 25 °C which is approximately a 5-fold decrease relative to that of the wild type (*k*_cat_ = 0.5 ± 0.2 s^–1^ at 25 °C^40^). It has been proposed that catalysis is rate-limited by subdomain opening in OdinAK^40^ and by interpreting the obtained catalytic parameters using this model, our data suggest that the rate constant of subdomain opening in the Ser74Gly variant is reduced compared to the wild type. Hence, the position of the local unfolding event in OdinAK appears to be an important hot spot for the kinetics of the open-to-closed transition. Since the native habitat temperature of archaeal species is significantly higher than 25 °C,^83^ we determined the catalytic efficiency of the OdinAK Ser74Gly variant at 65 °C to be 5.0 ± 0.2 s^–1^ (Figure 6C), which is in the same order of magnitude as the wild-type trimeric OdinAK (12 ± 4 s^–1^ at 65 °C^40^). Collectively, the results suggest that there is a network of interactions driving the conformational dynamics underlying catalysis in OdinAK and that the loss of one hydrogen bond to Arg2, as well as the destabilizing energetic contribution of an internal glycine (approximately 4 kJ mol^–1^ ^86^), is not sufficient to drive the conformational change of the S74-helix during subdomain closure. However, helical fraying as manifested here in the unfolding and translational changes in the S74-helix might still be a microscopic structural prerequisite, although we have showed with both structural and functional data that residue Ser74 is not the sole driver nor causative for subdomain closing in OdinAK.

**Figure 6:**
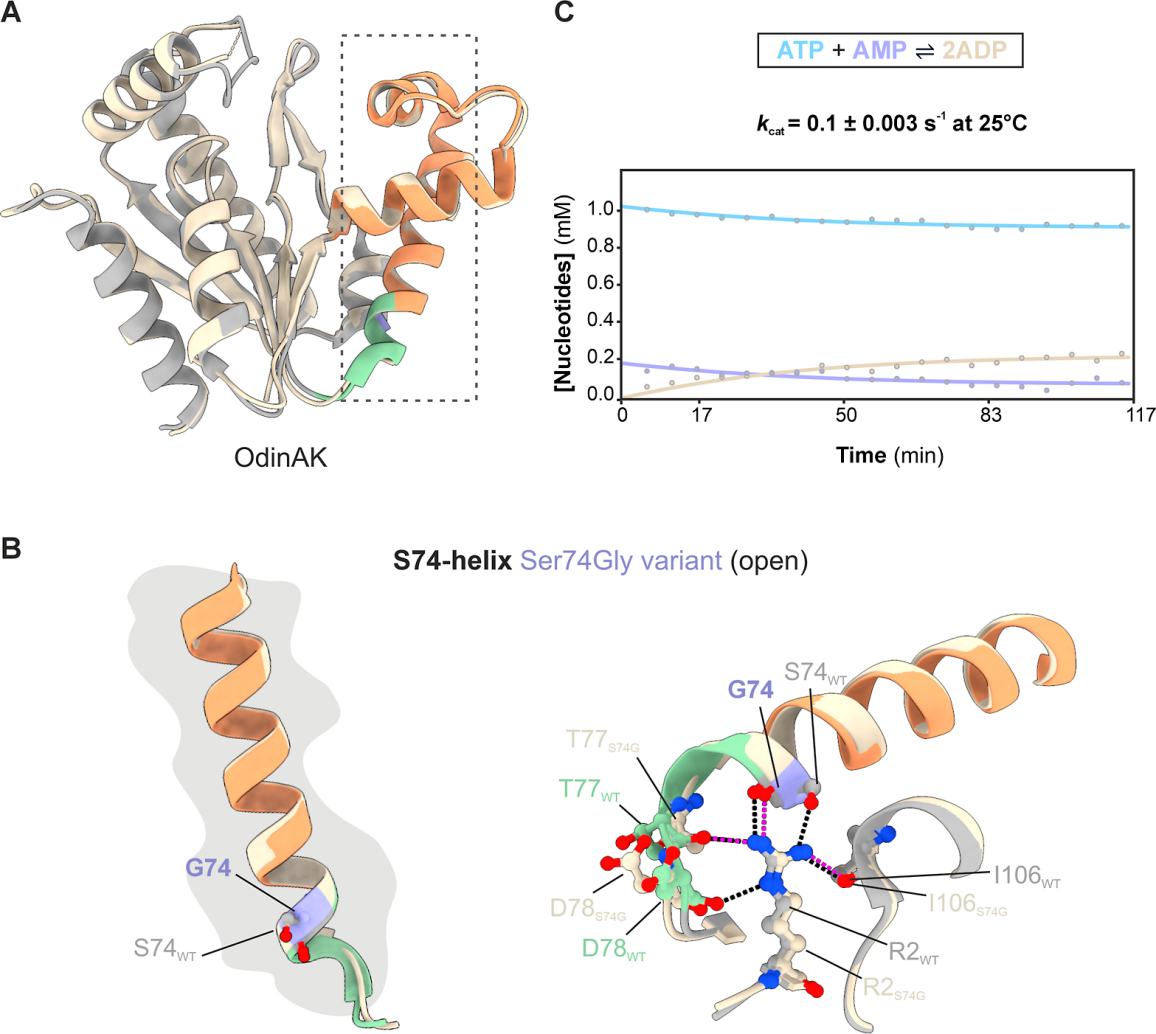
Structural and functional characterization of the OdinAK Ser74Gly variant. (A) Overlay of one monomer (chain A) of the OdinAK Ser74Gly variant (beige, PDB ID: 9R72 (this study)) and wild-type OdinAK (gray, PDB ID: 7OWH). The S74-helix in OdinAK is shown in an equivalent color scheme to Figure 5. (B) Zoom-in on an overlay of the S74-helix in the wild-type OdinAK (coloring as in panel A) and OdinAK Ser74Gly variant (beige) with the introduced glycine residue at position 74 colored purple (left panel). Hydrogen bonds between Arg2 and the native and Ser74Gly variant are shown in black and magenta dotted lines, respectively (right panel). (C) Quantification of the catalytic efficiency of the OdinAK Ser74Gly variant at 25 °C by a real-time ^31^P NMR assay. The catalytic parameter, *k*_cat_, was obtained by fitting the nucleotide concentrations to the rate equations described in reference 57.

### Structural Ordering and Helical Formation during the Open-to-Closed Transition in Human Adenylate Kinase

Having found that the plasticity of the termini of α-helices in the selected examples of bacterial and archaeal AKs is linked to enzymatic catalysis, we asked if we could identify related examples in a eukaryotic adenylate kinase. To this end, we manually inspected human adenylate kinase (hAK1), for which there exist deposited but unpublished crystallographic structures for both the apo state (PDB ID: 7DE3) and the Ap5A-bound state (PDB ID: 1Z83). Analysis of these structures shows that the entire catalytic loop and the C-terminal residues in the α-helix that directly precede the catalytic loop are not modeled in the apo state as they lack electron density (missing residues Gly133–Asp141). On the contrary, these residues are well-defined in the electron density in the Ap5A-bound state. This points toward a possible exploitation of structural plasticity mediated through helix formation during the transition from the substrate-free to substrate-bound and catalytically active states in hAK1. One question we wanted to address was if the structural ordering of this local region involves reduced flexibility of an already folded loop or if it involves folding of a disordered region. To avoid influence on folding from crystal packing contacts, we determined the degree of order in these states by quantification of NMR order parameters (*S* ^2^) in solution.^50,51^ Order parameters can be modeled from NMR spin relaxation rates (here ^15^N R1, R2 and ^1^H–^15^N steady-state NOE) and where the extremes zero and one represent complete disorder and order of the amide bond vector on the fast ps–ns time scale, respectively. The determined and fitted order parameters for hAK1 are shown in Figure 7A. Interestingly, the most notable features are low order parameters in the catalytic loop of the apo state. Since we are interested in the changes in the transition between apo and substrate-bound states, we define Δ*S* ^2^.^87^ This parameter corresponds to the change in order upon moving from the apo to the Ap5A-bound state, where a positive value signifies an increased degree of order with Ap5A. To identify statistically significant values, we used a trimmed average^54^ plus three standard deviations as a threshold. A display of Δ*S* ^2^ versus primary sequence (Figure 7B) shows that there exists one continuous stretch of residues with a significant change. This stretch corresponds to the catalytic loop, where the residues become more ordered in the Ap5A-bound and closed hAK1 conformation. Residues with an increased degree of order are displayed on the hAK1 structure in Figure 7C. Notably, one helical turn in the α-helix at the N-terminus of the catalytic loop (residues Thr126–Lys131 in the apo structure) became ordered upon Ap5A binding and now comprises an additional helical turn including residues Arg132–Thr135. Therefore, in agreement with the crystallographic structures, the transition from the apo to the bound hAK1 state in solution is accompanied by helix formation in the C-terminus of the helix preceding the catalytic loop. This finding suggests that the catalytic loop is not preformed but flexible in the apo state, and undergoes a coupled folding and binding event^22,88^ during which substrate binding is a necessary requirement for formation of the catalytically competent active site structure in hAK1.

**Figure 7:**
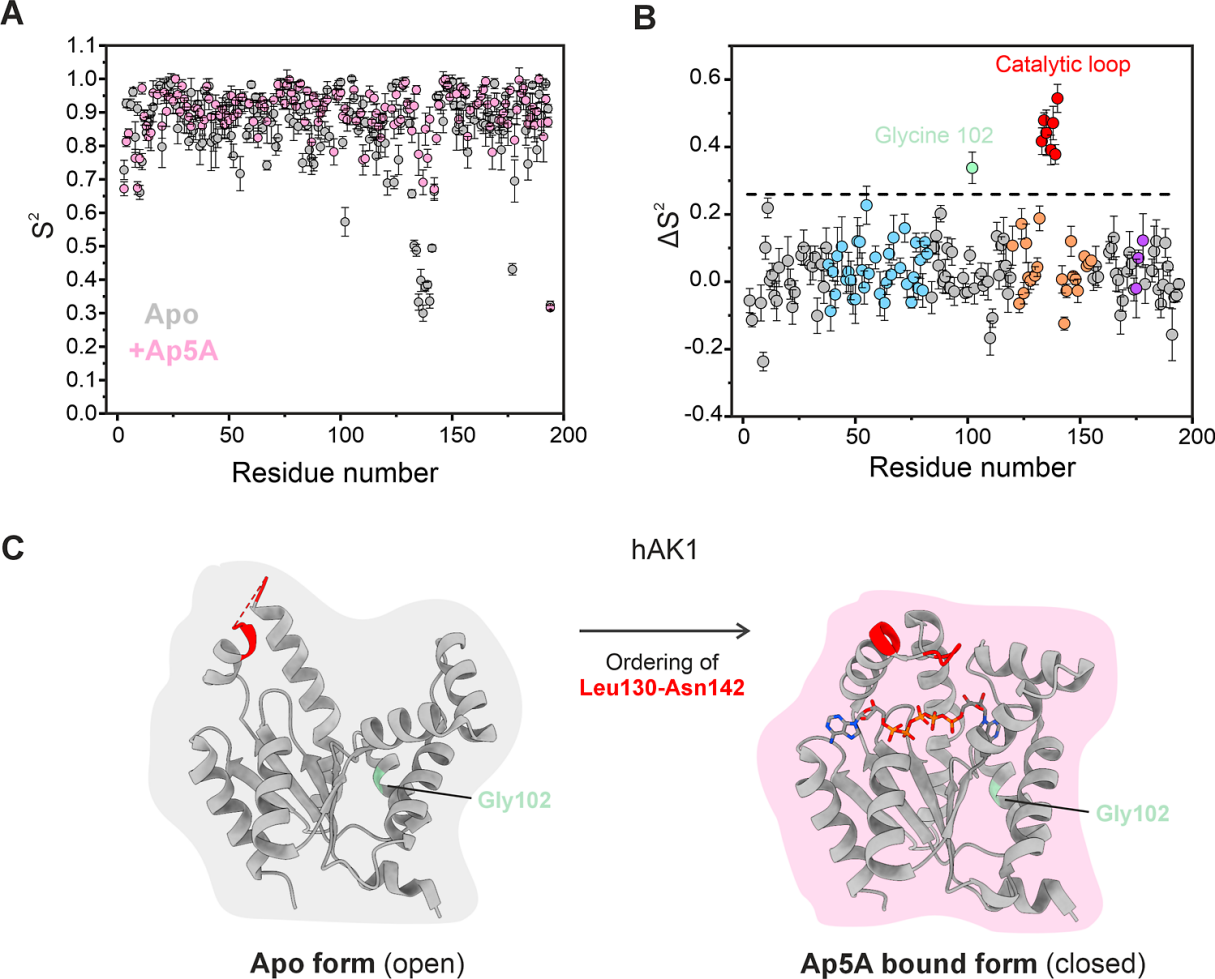
Ordering of the catalytic loop in hAK1 upon binding of Ap5A. (A) Order parameters (*S* ^2^) for hAK1 in apo (gray) and Ap5A-bound states (pink) displayed versus the primary sequence. The shown values of *S* ^2^ are obtained from data acquired at a 850 MHz field based on spin relaxation rates shown in Figure S10. The root-mean-square error associated with fits of each value of *S* ^2^ with the model-free approach^50,51^ is indicated on each bar graph. (B) Difference in *S* ^2^ (Δ*S* ^2^) between apo and Ap5A-bound states of hAK1 displayed against the primary sequence. The black dotted line corresponds to the trimmed mean average of *S* ^2^ plus three standard deviations and serves as a threshold value. The color-coding is as follows: residues corresponding to the ATPlid in orange, AMPbd in blue, the catalytic loop in red, and the selectivity loop in purple. (C) Structural mapping of the residues that display significant ordering (Δ*S* ^2^ above threshold) upon Ap5A binding in hAK1 (PDB ID: 7DE3 (apo) and PDB ID: 1Z83 (Ap5A-bound)). The involved residues Leu130–Asn142, shown in red, include the catalytic loop. The position of Gly102 (green) is shown with an arrow. This residue is not part of a continuous stretch of residues with perturbed order and is therefore not discussed further.

## Conclusions

Conformational dynamics is a key aspect underlying the function of enzymes in order to, for instance, accommodate binding and release of substrates^31^ and to assemble active sites.^35^ A linkage between the dynamics required for ligand binding and release and catalysis has also been discovered.^13^ Macroscopic models that account for conformational dynamics include induced fit^14^ and conformational selection models^17^ or combinations of these two extremes.^18^ It has been shown that common microscopic mechanisms used by proteins to support conformational changes are rotations around hinge regions,^89^ and this concept has been applied to explain the subdomain closure in AK.^42^ Here, we have addressed the underlying structural basis being utilized to support conformational changes in α-helices in AKs, and we confirm that the intrinsic low stabilities at the termini of α-helices are ideal hot spots to support conformational changes. We investigated various flavors of structural changes at the termini of α-helices during the open to closed structural transition in the enzymes studied. These changes could be described as (1) helical bending between folded structures of an α-helix (AK_eco_ case); (2) local disordering of the C-terminal end of an α-helix to accommodate closure of the AMP-binding subdomain (OdinAK case); and (3) local folding of the C-terminus of an α-helix with subsequent ordering of the catalytic loop (hAK1 case). From a functional perspective, perturbation of these structural mechanisms could influence the catalytic parameters in diverse manners, resulting in either unaltered or significantly altered catalytic capacities. As an example, in AK_eco_ with the Glu114Ala replacement, we observed an increase in the specificity constant, a change consistent with release of the substrate as the limiting event in the reaction cycle.^90^ On the other hand, the Lys47Ala replacement led to a less stable protein with a more dynamic K47-helix and increased catalytic activity. The Ser74Gly replacement in OdinAK was silent with respect to *k*_cat_. Hence, the local unfolding reaction in OdinAK, although necessary for the closing of the AMPbd, cannot be provoked by the replacement of Ser74 with a helix-breaking glycine residue. This fact is reconciled with the structural stability originating from the folded structure, which in turn outweighs the destabilizing effect caused by the introduction of a glycine residue at position 74. Two of the investigated cases involve local folding (hAK1) and local unfolding (OdinAK) events on the open-to-closed conformational transition. Coupled folding and binding events are relatively common in biology and have been observed, as example, for protein–protein interactions,^22^ protein–ion interactions,^91^ and protein–membrane interactions.^88^ In the case of hAK1, we discovered that a coupled folding and binding reaction is linked to ordering of the unfolded catalytic loop and is consequently of importance for the assembly of the active site. Local unfolding reactions linked to biology, as we observed for OdinAK, seem to be less common. However, it has also been observed for other AKs^25,26^ as well as the transition between the prepore and pore state of the TcA toxin from *P. luminescens*.^92^ Microscopic structural fluctuations and plasticity, as we have probed here in terms of helical plasticity and unfolding/folding events, are also found in other proteins. Examples include transitions in terms of helix rotations and bends,^93^ helical unwinding, reformation and translations,^94^ and N-capping.^95^ The concept of “cracking” has been described as a key step in ATP hydrolysis in the catalytic domain of a P-ATPase.^96^ Other types of structural transitions centered on α-helices have also been linked to function, such as regular helix to π-helix transition^97^ in the function of cytochrome c oxidase.^98^ In light of the seemingly vast expression of helical plasticity that we and others have explored, it appears possible that termini of α-helices can be utilized as hot spots for conformational change, which is likely rooted in the energetic landscape of the α-helix itself. Ideal α-helices are stabilized through short-range hydrogen bonding networks between backbone atoms^99^ and further stabilized by capping interactions at their N- and C-termini.^100^ These local interactions, contrasting the long-range interactions observed in β-strands, have low contact order^101^ and consequently carry a limited entropic penalty for their folding and unfolding. The simplicity of their local interactions has allowed development of models of helix formation from polymer physics, such as the Zimm–Bragg model that explains helix formation with only two parameters: nucleation and propagation.^102^ The propagation parameter implies that helices can fold and unfold, particularly at their N- and C-termini (fraying), a feature that also has been demonstrated experimentally.^103,104^ These biophysical properties of α-helices and, in particular, N- and C-terminal fraying make them ideal as hot spots for local structural changes during conformational changes in proteins. Taken together, the findings presented here add additional examples to the rich variety of microscopic events concentrated at the termini of α-helices possibly enabling macroscopic conformational changes in proteins.

## Supplementary Material



## Data Availability

Chemical shift assignments were deposited in the Biological Magnetic Resonance Data Bank (BMRB) with the following accession IDs: hAK1 apo (52487), hAK1 ADP-bound (52487), and Ap5A-bound (52489) states, all in MES buffer at pH 5.5. The atomic coordinates and structure factors for the presented crystallographic structures were deposited in the Protein Data Bank (PDB), Research Collaboratory for Structural Bioinformatics, Rutgers University, New Brunswick, NJ, with the following accession IDs: Crystal structure of the *E. coli* AK E114A mutant in complex with inhibitor Ap5A (9R71), crystal structure of the *E. coli* AK K47A mutant in complex with inhibitor Ap5A (9R6U), and crystal structure of the *Odinarchaeota* AK (OdinAK) S74G mutant (9R72).

## Notes

Structure of OdinAK in the trimeric arrangement; Michaelis–Menten kinetics of the AK_eco_ enzyme variants; thermal stabilities of the AK_eco_ variants; HSQC spectra of the AK_eco_ Glu114Ala variant; HSQC spectra of the AK_eco_ Lys47Ala variant; projection analysis of AK_eco_ Glu114Ala in the Ap5A-bound state; projection analysis of AK_eco_ Glu114Ala in the apo state; projection analysis of AK_eco_ Lys47Ala in the Ap5A-bound state; projection analysis of AK_eco_ Lys47Ala in the apo state; backbone amide ^15^N–^1^H heteronuclear NOEs of hAK1 in apo, ADP, and Ap5A states; data collection and refinement statistics of the determined crystallographic structures; thermal stability data on the AK_eco_ variants; and data on the projection analyses of the AK_eco_ variants (PDF)
