# Supplementary material for: Exploring Helical Fraying Linked to Dynamics and Catalysis in Adenylate Kinase
Source: Biochemistry. 2025 Oct 3;64(20):4281–95. doi: 10.1021/acs.biochem.5c00306 (PMC12548092; doi:10.1021/acs.biochem.5c00306)
Supplement: Supplementary file 1 [file bi5c00306_si_001.pdf]

Supplementary Information

Exploring Helical Fraying Linked to Dynamics and Catalysis in Adenylate Kinase

Jonna Mattsson[1], Chanrith Phoeurk[1,2], Léon Schierholz[1,3], Ameeq Ul Mushtaq[1,4], Jhon Alexander Rodriguez Buitrago[1,5], Per Rogne[1], A. Elisabeth Sauer-Eriksson*[1] & Magnus Wolf-Watz*[1]

[1]Department of Chemistry, Umeå University, 901 87 Umeå, Sweden

[2]Department of Bio-Engineering, Royal University of Phnom Penh, 120404 Phnom Penh, Cambodia

[3]Department of Molecular Biology, Umeå University, 901 87 Umeå, Sweden

[4]Department of Pharmacology, Northwestern University, Feinberg School of Medicine, Chicago, 60611 Illinois, United States

[5]Department of Chemistry, Universidade Nova de Lisboa, Science and Technology Faculty, 2829-516 Caparica, Portugal

Correspondence to: elisabeth.sauer-eriksson@umu.se and magnus.wolf-watz@umu.se

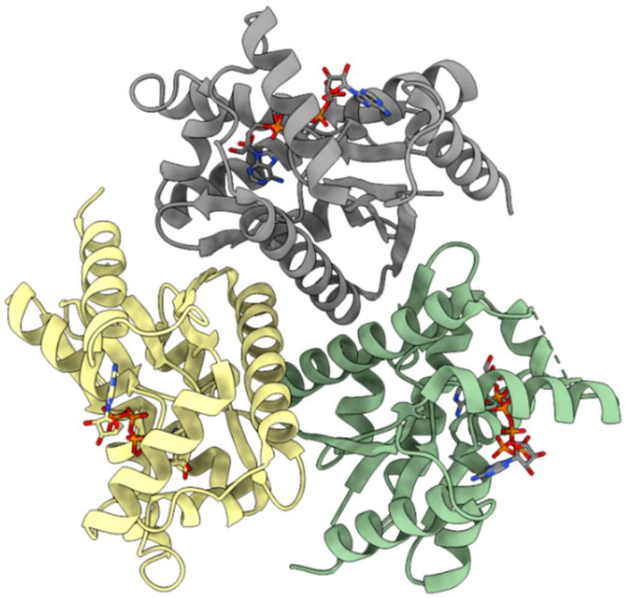

Figure S1. Biological trimer of OdinAK in complex with Ap5A. The three monomers (grey, yellow and green) assemble into the biological trimer via the trimerization helices (PDB ID: 7OWE[1]).

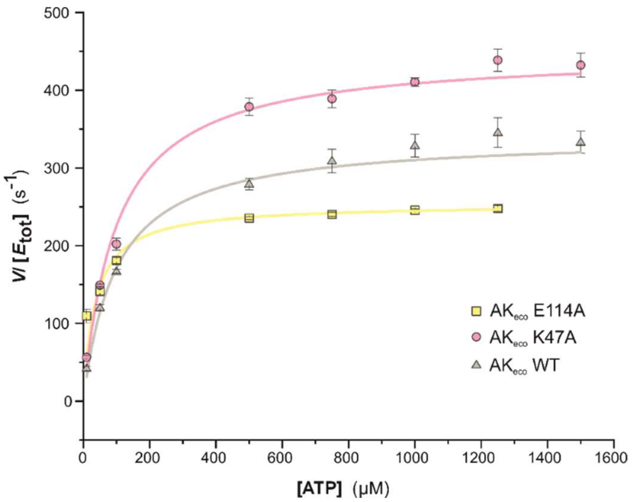

Figure S2. Enzymatic activities of the AK_{eco} enzyme variants. The normalized reaction velocities ($V/[E_{tot}]$) are shown as a function of varying ATP concentrations. The solid lines are fits to the Michaelis-Menten equation for respective AK_{eco} variant, Glu114Ala (yellow), Lys47Ala (pink) and wild type (gray). Error bars show standard deviations (SD) derived from technical triplicates.

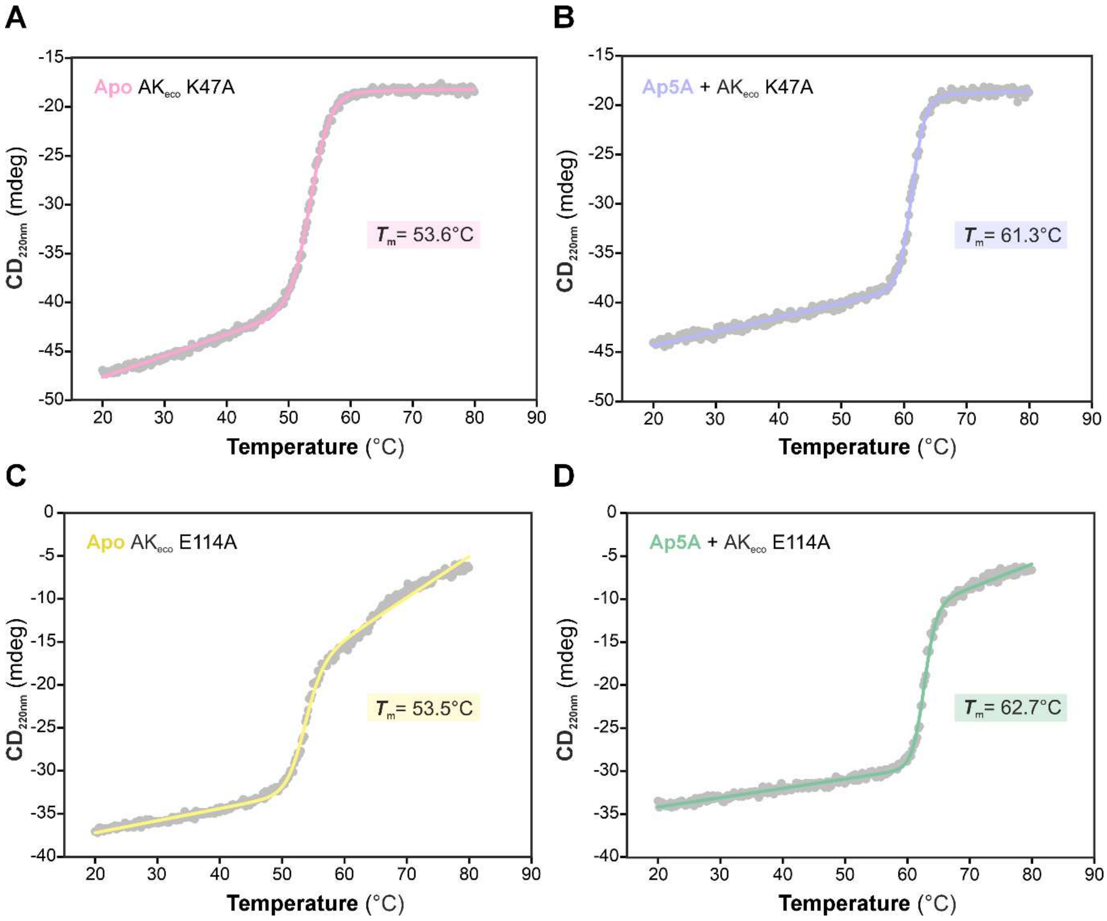

Figure S3. Thermal stabilities of the AK_{eco} variants Glu114Ala and Lys47Ala. Protein stabilities were quantified by thermal unfolding experiments, where the CD signal at 220 nm as a function of temperature was followed. The melting points (T_m) were determined by fitting the obtained data to a two-state transition (solid lines). (**A**) Apo AK_{eco} Lys47Ala in pink and Ap5A-bound in purple (**B**); and (**C**) Apo AK_{eco} Glu114Ala in yellow and Ap5A-bound in turquoise (**D**).

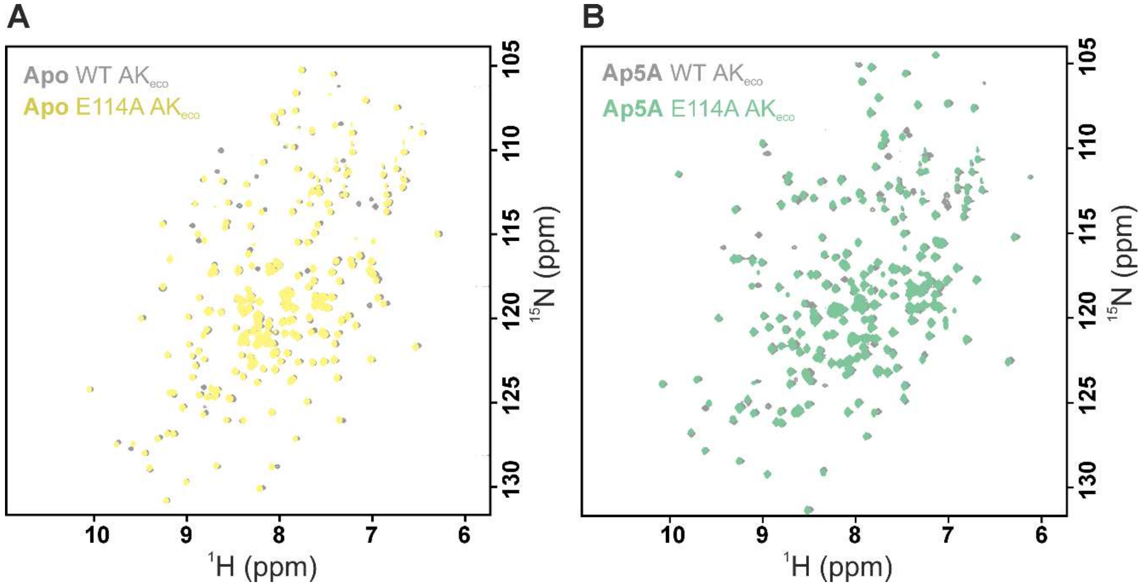

Figure S4. ¹H-¹⁵N-HSQC of the AK$_{eco}$ Glu114Ala variant. (**A**) Overlay of AK$_{eco}$ Glu114Ala open (apo) in yellow and wild type in gray. (**B**) Overlay of closed (Ap5A-bound) states of AK$_{eco}$ Glu114Ala (turquoise) and wild type (gray).

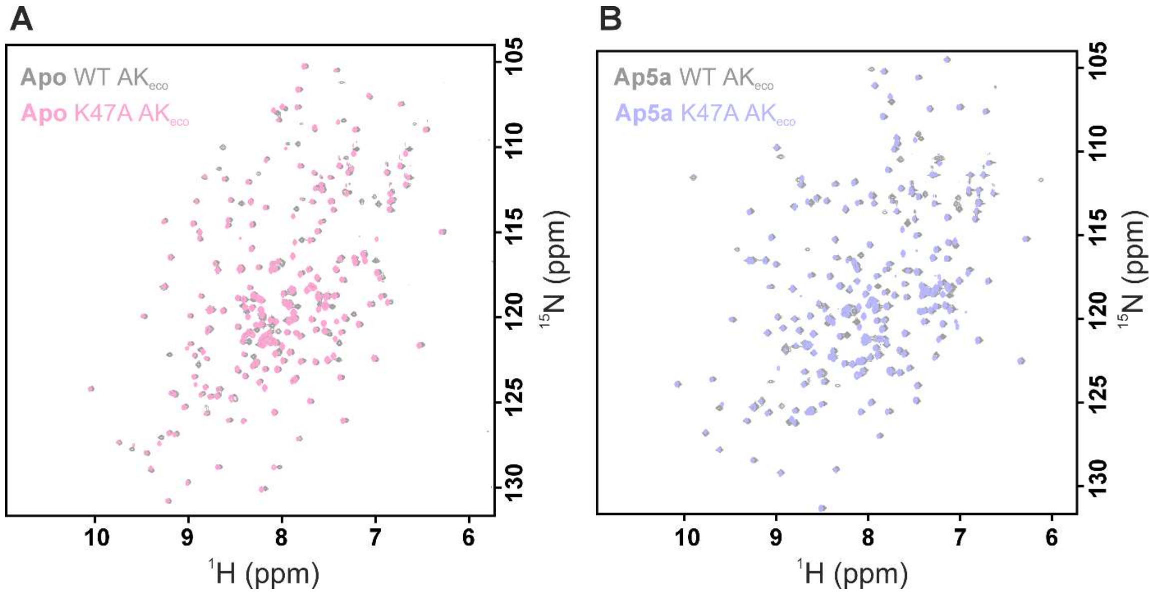

Figure S5. ¹H-¹⁵N-HSQC of the AK$_{eco}$ Lys47Ala variant. (A) Overlay of AK$_{eco}$ Lys47Ala open (apo) in pink and wild type in gray. (**B**) Overlay of closed (Ap5A-bound) states of AK$_{eco}$ Lys47Ala and wild type, purple and gray, respectively.

Projection analysis of AK_{eco} E114A (Ap5A) using WT (apo) as the reference and the WT (Ap5A) as the comparing vector

A projection analysis showing to which degree the replacement of the glutamic acid at position 114 with an alanine (AK_{eco} Glu114Ala) changes the propensity for AK_{eco} to close upon binding to Ap5A. Data on the projection analysis can be found in Table S3.

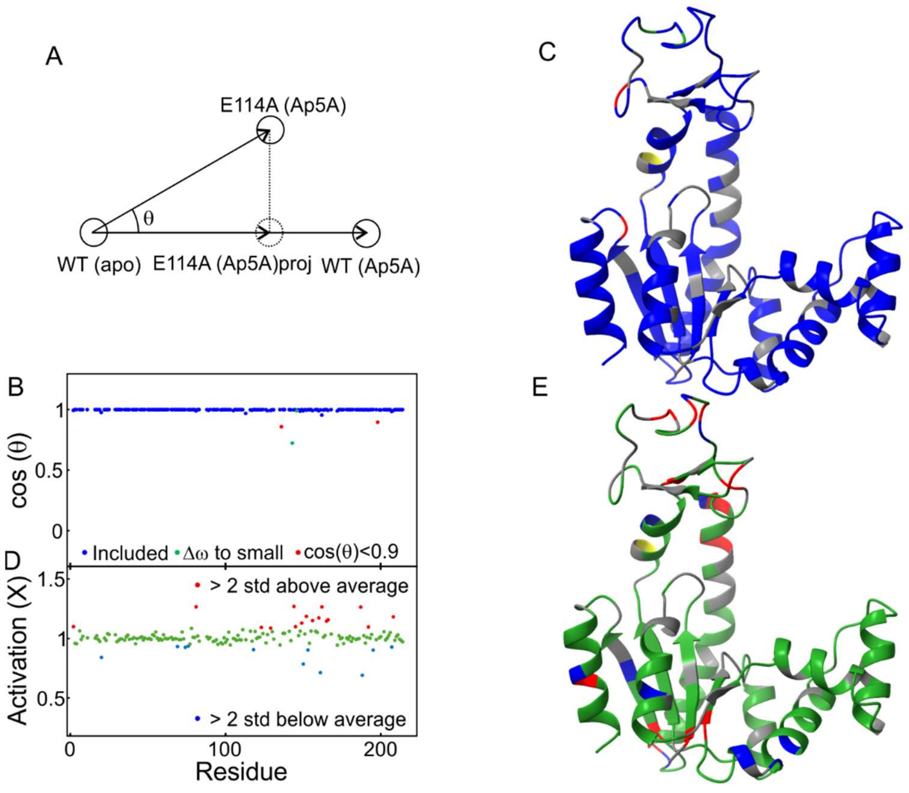

Figure S6. Graphical representation of the projection analysis of AK_{eco} E114A (Ap5A). (**A**) A schematic illustration of the projection analysis, the activation (X) is the quotient of the magnitude of the vector between WT (apo) and E114A (Ap5A)proj and the vector between WT (apo) and WT (Ap5A). In (**B**) a cos(θ) of close to 1 indicates that the directions of the two vectors are the same and that most of the chemical shifts determined are due to the same process, in this case the closing of AK_{eco}. The blue markers in (**B**) indicate amino acid residues included in the projection analysis and the green markers indicate residues where the chemical shift differences between the states are less than two standard deviations larger than the average of the difference between the WT and the mutant in regions not affected by the mutation. In (**C**) these categories are plotted on the structure of open AK_{eco} (PDB ID: 4AKE) using the same colors as in (**B**). (**D**) Shows the activation of each of the amino acid residues split into three categories: red more than two standard deviations above the average, green within two standard deviations of the average, and blue more than two standard deviations below the average. (**E**) Shows the same categories plotted on the open AK_{eco} structure.

Projection analysis of AK_{eco} E114A (apo) using WT (apo) as the reference and the WT (Ap5A) as the comparing vector

The following projection analysis shows to which degree the replacement of the glutamic acid at position 114 with an alanine (AK_{eco} Glu114Ala) shifts the conformation of AK_{eco} towards the closed conformation as represented by wild type AK_{eco} bound to Ap5A. The data on the projection analysis can be found in Table S3.

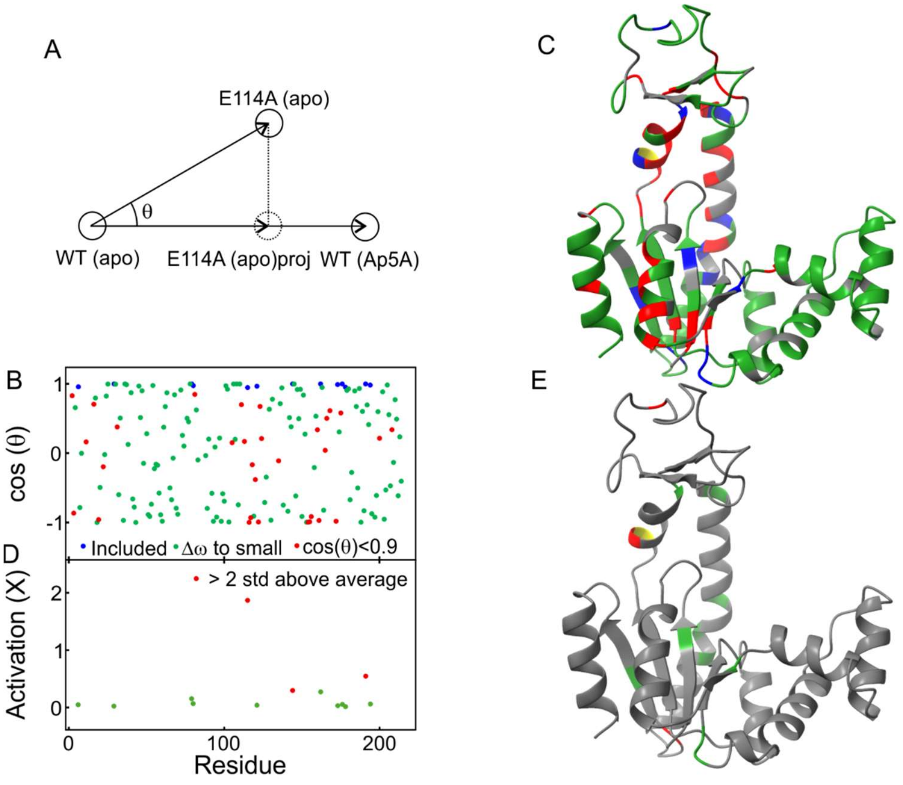

Figure S7. Graphical representation of the projection analysis of AK_{eco} E114A (apo). (**A**) Schematic illustration of the projection analysis, the activation (X) is the quotient of the magnitude of the vector between WT (apo) and E114A (apo)proj and the vector between WT (apo) and WT (Ap5A). In (**B**) a cos(θ) of close to 1 indicates that the directions of the two vectors are the same and that most of the chemical shifts determined are due to the same process, in this case the closing of AK_{eco}. The blue markers in (**B**) indicate amino acid residues included in the projection analysis and the green markers indicate residues where the chemical shift differences between the states are less than two standard deviations larger than the average of the difference between the WT and the mutant in regions not affected by the mutation. In (**C**) the categories are plotted on the structure of open AK_{eco} (PDB ID: 4AKE) using the same colors as in (**B**). (**D**) Shows the activation of each of the amino acid residues split into three categories: red more than two standard deviations above the average, green within two standard deviations of the average, and blue more than two standard deviations below the average, of which there were none. Since residue 115 fulfilled the criteria to be included even if it is the neighboring residue to the replacement, this residue is marked as included in the analysis, however, the average and standard deviation has been calculated without it. (**E**) Shows the same categories plotted on the open AK_{eco} structure.

Projection analysis of AK$_{eco}$ K47A (Ap5A) using WT (apo) as the reference and the WT (Ap5A) as the comparing vector

The following projection analysis shows to which degree binding of Ap5A to the AK$_{eco}$ Lys47Ala variant induces the same chemical shifts as the binding of Ap5A to the wild type. The data on the projection analysis can be found in Table S3.

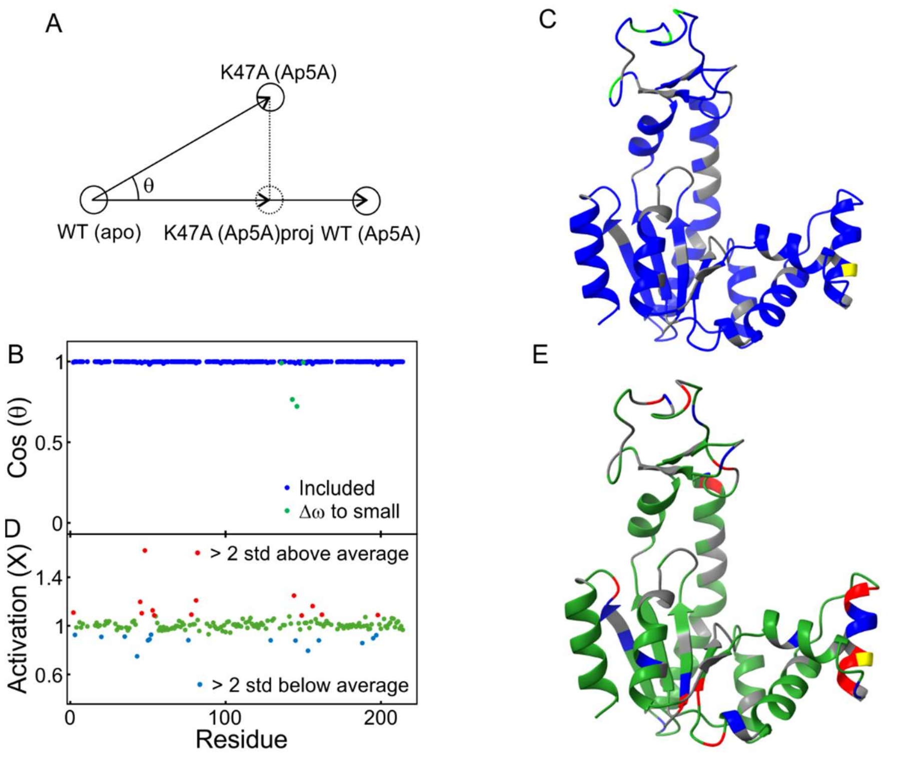

Figure S8. Graphical representation of the projection analysis of AK$_{eco}$ K47A (Ap5A). (**A**) Shows a schematic illustration of the projection analysis, the activation (X) is the quotient of the magnitude of the vector between WT (apo) and K47A (Ap5A)proj and the vector between WT (apo) and WT (Ap5A). In (**B**) a cos(θ) of close to 1 indicates that the directions of the two vectors are the same and that most of the chemical shifts determined are due to the same process, in this case the closing of AK$_{eco}$. The blue markers in (**B**) indicate amino acid residues included in the projection analysis and the green markers indicate residues where the chemical shift differences between the states are less than two standard deviations larger than the average of the difference between the WT and the mutant in regions not affected by the mutation. In (**C**) the categories are plotted on the structure of open AK$_{eco}$ (PDB ID: 4AKE) using the same colors as in (**B**). (**D**) Shows the activation of each of the amino acid residues split into three categories: red more than two standard deviations above the average, green within two standard deviations of the average, and blue more than two standard deviations below the average. (**E**) Shows the same categories plotted on the open AK$_{eco}$ structure.

Projection analysis of AK_{eco} K47A (apo) using WT (apo) as the reference and the WT (Ap5A) as the comparing vector

The projection analysis shows to which degree the replacement of the lysine at position 47 with an alanine (AK_{eco} Lys47Ala) shifts the conformation of AK_{eco} towards the closed conformation represented by wild type AK_{eco} bound to Ap5A. Data on the projection analysis can be found in Table S3.

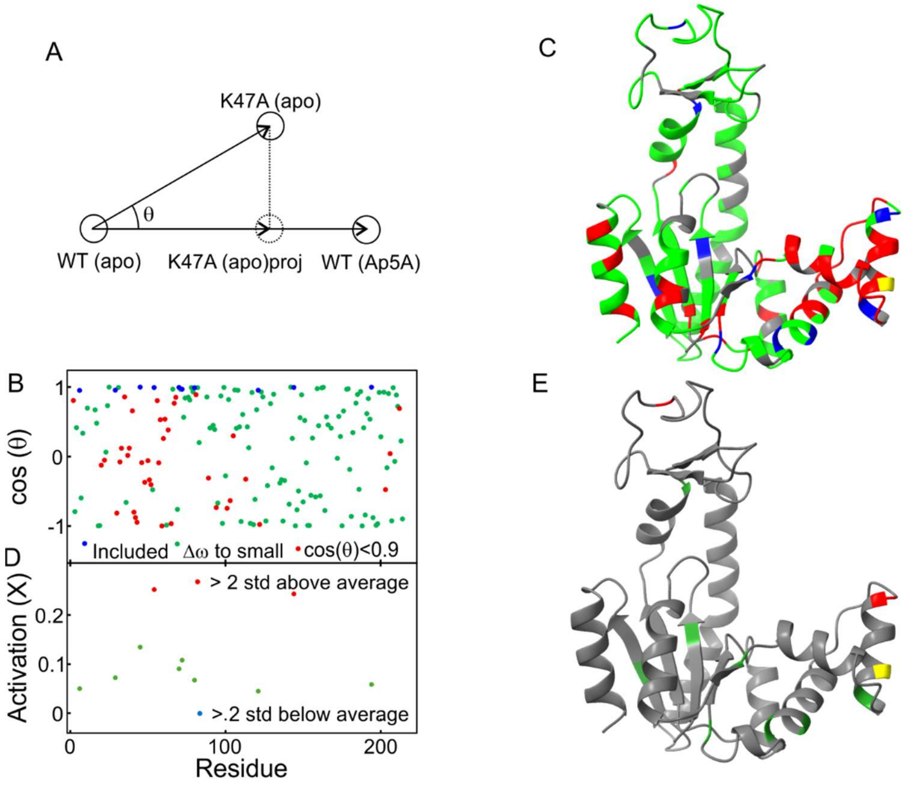

Figure S9. Graphical representation of the projection analysis of AK_{eco} (apo). (**A**) Shows a schematic illustration of the projection analysis, the activation (X) is the quotient of the magnitude of the vector between WT (apo) and K47A (apo)proj and the vector between WT (apo) and WT (Ap5A). In (**B**) a cos(θ) of close to 1 indicates that the directions of the two vectors are the same and that most of the chemical shifts determined are due to the same process, in this case the closing of AK_{eco}. The blue markers in (**B**) indicate amino acid residues included in the projection analysis and the green markers indicate residues where the chemical shift differences between the states are less than two standard deviations larger than the average of the difference between the WT and the mutant in regions not affected by the mutation. In (**C**) the categories are plotted on the structure of open AK_{eco} (PDB ID: 4AKE) using the same colors as in (**B**). (**D**) Shows the activation of each of the amino acid residues split into three categories: red more than two standard deviations above the average, green within two standard deviations of the average, and blue more than two standard deviations below the average. (**E**) Shows the same categories plotted on the open AK_{eco} structure.

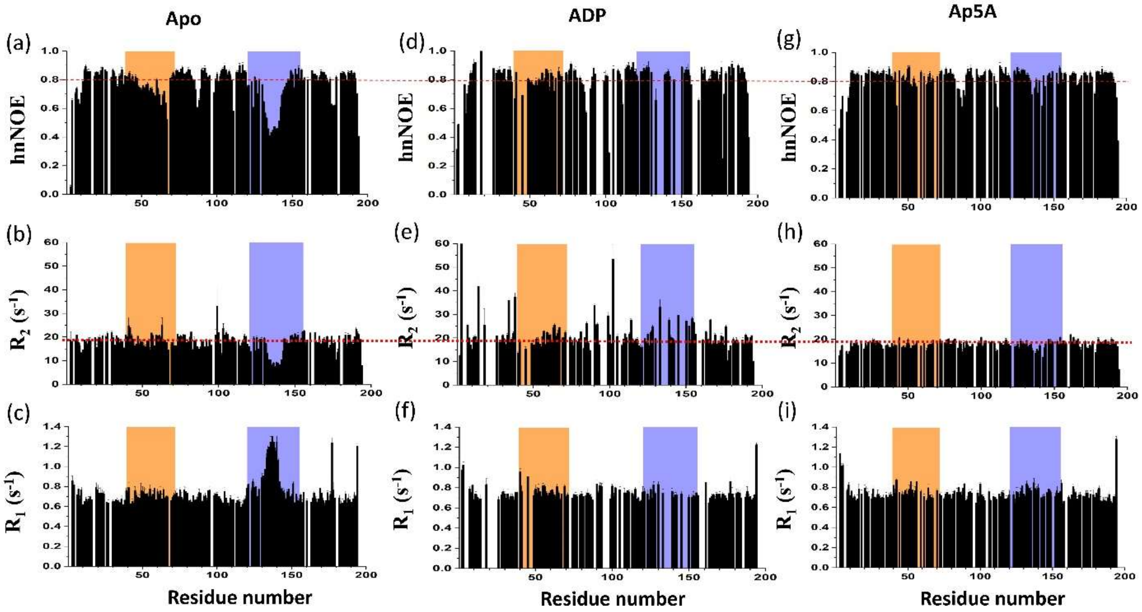

Figure S10. Plot of backbone amide ^{15}N-^{1}H heteronuclear NOEs, R_2 and R_1 relaxation parameters along the residue numbers for human Adenylate Kinase 1 (hAK1) apo state, ADP state and Ap5A states. First column shows the (a) hnNOE values (b) R_2 values and (c) R_1 values for apo state plotted along the hAK1 residue number. Second column shows the (d) hnNOE values (e) R_2 values and (f) R_1 values for ADP state of hAK1 plotted along the residue number and third column shows the (g) hnNOE values (h) R_2 values and (i) R_1 values for Ap5A state plotted along the hAK1 residue number. ^{15}N-^{1}H heteronuclear NOEs, R_2 and R_1 relaxation parameters are obtained from data acquired at 850 MHz field. Error bars are placed on top of the bar graphs. AMP-Lid and ATP-Lid domains are highlighted in orange and light blue color respectively.

Table S1. Data collection and refinement statistics for the solved structure of the AK enzymes included in the study.

**Data-collection statistics**	**AK_{eco} E114A + Ap5A**	**AK_{eco} K47A + Ap5A**	**OdinAK S74G**
Synchrotron/Beamline	ESRF/ID29	ESRF/ID30A_3	ESRF/ID30A-3
Wavelength (Å):	0.8731	0.9677	0.9677
Space group:	$P2_12_12$	$P2_122_1$	$P2_1$
Unit-cell parameters (Å,°)	72.96, 84.52, 78.93<br>90.0, 90.0, 90.0	73.76, 79.06, 83.11<br>90.0, 90.0, 90.0	77.64, 77.00, 115.86<br>90.0, 95.87, 90.0
Resolution limits[a] (Å)	57.69 -1.61 (1.64 -1.61)	41.56-1.77 (1.83-1.77)	41.33-3.58 (3.71-3.58)
No. of unique reflections	63456 (3017)	45314 (3309)	16240 (1615)
Multiplicity	13.5 (13.1)	3.9 (3.2)	7.6 (7.4)
Completeness (%)	99.7 (96.8)	94.4 (70.0)	99.9 (100.0)
R_{merge}	0.104 (2.496)	0.056 (0.890)	0.512 (2.151)
R_{PIM}	0.029 (0.707)	0.048 (0.783)	0.230 (1.055)
$<I/\sigma (I)>$	14.2 (1.1)	12.4 (1.3)	3.3 (0.7)
CC1/2	0.999 (0.510)	0.998 (0.324)	0.963 (0.374)
Wilson B-factor ($Å^2$)	21.0	27.2	99.1
R factor (%)	0.198 (0.331)	0.192 (0.451)	0.247 (0.379)
R free (%)	0.244 (0.388)	0.228 (0.471)	0.274 (0.372)
No. of protein atoms	3318	3370	9299
No. of water molecules	369	401	2
No. of ligand atoms	114	178	5
Bond length (Å)	0.005	0.008	0.007
Bond angles (°)	0.85	1.10	0.95
Clash score	3.5	1.7	5.1
Ramachandran: Residues in most favored regions (%)	99.5	99.1	97.8
Ramachandran: Residues in disallowed regions (%)	0.0	0.0	0.0
Average B-factor protein ($Å^2$)	38.1	32.5	93.5
Average B-factor ligands ($Å^2$)	32.2	23.3	94.4
Average B-factor solvent ($Å^2$)	39.1	38.1	48.1
PDB code	9R71	9R6U	9R72

[a]Values in parenthesis are for the high-resolution shell. Data were collected from one crystal. Resolution limits were determined by applying a cut-off based on the mean intensity correlation coefficient of half-datasets, CC1/2, at the high-resolution bin (>0.30).

Table S2. Thermal stabilities of AK_{eco} variants.

	Apo (T_m, °C)	Ap5A (T_m, °C)
Wild type[a)]	57.0 ± 0.2	64.5 ± 0.2
Lys47Ala	53.6 ± 0.0 [b)]	61.5 ± 0.2
Glu114Ala	53.6 ± 0.1	62.7 ± 0.0

[a)] Thermal stability data of wild type AK_{eco} from Ådén et al.[2] (not this study).
[b)] Errors are estimated from fits of the data to a two-state transition (fits are found in Figure S3) from two technical replicates.

Table S3. Data on the projection analyses of the AK_{eco} variants in apo and Ap5A bound states.

	E114A (apo)[a)]	E114 (Ap5A)[b)]	K47A (apo)[c)]	K47A (Ap5A)[d)]
Total residues in AK_{eco}	214	214	214	214
Not fully assigned	44	44	44	43
Too small chemical shift	126	2	121	4
Too large angle (Cos(θ)<0.9)	31	2	39	0
Residues in analysis	13 (including 115)	166	10	167
Average X	0.134 ± 0.154	1.012 ± 0.074	0.112 ± 0.072	1.007 ± 0.077

[a)] Projection analysis of AK_{eco} E114A (apo) using WT (apo) as the reference and the WT (Ap5A) as the comparing vector.
[b)] Projection analysis of AK_{eco} E114A (Ap5A) using WT (apo) as the reference and the WT (Ap5A) as the comparing vector.
[c)] Projection analysis of AK_{eco} K47A (apo) using WT (apo) as the reference and the WT (Ap5A) as the comparing vector.
[d)] Projection analysis of AK_{eco} E114A (Ap5A) using WT (apo) as the reference and the WT (Ap5A) as the comparing vector.

References

1. Verma A, Aberg-Zingmark E, Sparrman T, Mushtaq AU, Rogne P, Grundstrom C, et al. Insights into the evolution of enzymatic specificity and catalysis: From Asgard archaea to human adenylate kinases. Sci Adv. 2022;8(44):eabm4089.

2. Ådén J, Verma A, Schug A, and Wolf-Watz M. Modulation of a Pre-existing Conformational Equilibrium Tunes Adenylate Kinase Activity. J. Am. Chem. Soc. 2012:134(40):16562-16570.
